# Highly expressed RPLP2 inhibits ferroptosis to promote hepatocellular carcinoma progression and predicts poor prognosis

**DOI:** 10.1186/s12935-023-03140-0

**Published:** 2023-11-18

**Authors:** Jiaxing Guo, Meiyuan Huang, Shuang Deng, Haiyan Wang, Zuli Wang, Bokang Yan

**Affiliations:** 1 Department of Hematology, Zhuzhou Hospital Affiliated to Xiangya School of Medicine, Central South University, Zhuzhou, 412007 Hunan China; 2 Department of Pathology, Zhuzhou Hospital Affiliated to Xiangya School of Medicine, Central South University, Zhuzhou, 412007 Hunan China; 3 Academy of Biomedical Engineering, Kunming Medical University, Kunming, 650500 Yunnan China; 4 Center for Tissue Engineering and Stem Cell Research, Guizhou Medical University, Guiyang, 550025 Guizhou China

**Keywords:** HCC, Biomarker, RPLP2, GPX4, Ferroptosis, Prognosis, Bioinformatics

## Abstract

**Background:**

RPLP2, an integral part of ribosomal stalk, plays an important role in the tumorigenesis of various cancers. However, its specific effect on HCC remains elusive.

**Methods:**

TCGA, GTEx, HCCDB, HPA, UALCAN, MethSurv, TISIDB, K–M plotter, FerrDb, RNAactDrug, STRING, Cytoscape and R studio were conducted for bioinformatics analysis. RPLP2 expression level in HCC was verified by IHC and western blot. IHC was used to demonstrate the immune cell infiltration. Functional experiments including CCK8, transwell and colony formation assays, and nude mice xenograft model were performed for in vitro and in vivo validation. Western blot, IHC, CCK8 assay and detection of GSH and lipid ROS were adopted to determine the effect of RPLP2 on the ferroptosis of HCC cells.

**Results:**

Here, we demonstrate that elevated level of RPLP2 is strongly associated with advanced clinicopathologic features, and predicts poor prognosis of HCC patients. Additionally, DNA methylation level of RPLP2 decreases in HCC, and significantly correlates with patients outcome. Moreover, high RPLP2 expression level is linked closely to the unfavorable immune infiltration. Most importantly, RPLP2 positively associates with ferroptosis suppressor GPX4, and inhibition of RPLP2 could lead to the acceleration of ferroptosis to suppress tumor progression of HCC. Last, drug sensitivity analysis predicts many drugs that potentially target RPLP2.

**Conclusion:**

Together, our study reveals previous unrecognized role of RPLP2 in HCC, and provides new regulatory mechanism of ferroptosis, indicating RPLP2 may be a novel therapeutic target for HCC.

**Supplementary Information:**

The online version contains supplementary material available at 10.1186/s12935-023-03140-0.

## Introduction

Liver cancer is a highly malignant tumor with sixth in incidence and third in mortality among cancers globally [1]. Hepatocellular carcinoma (HCC) which accounts for 90% of liver cancer cases usually evolves from viral hepatitis C or B [2]. Although the survival of HCC patients could benefit from effective treatment due to the in-depth study of the biological and environmental mechanisms underlying HCC occurrence and progression, several areas, including high metastasis and recurrence rates of HCC, limited effective clinical options and bad performance of surgical treatment on advanced stage of HCC still need to be improved [3–5]. Thus, identification of novel molecular markers and effective prognostic signatures is desperately needed for improving the prognosis of HCC patients.

Ferroptosis, a newly defined form of regulated cell death, characterized by the accumulation of reactive oxygen species (ROS), is mainly induced by the intracellular iron catalytic activity and lipid peroxidation [6, 7]. Mechanistically, the cystine/glutamate antiporter system Xc−/glutathione (GSH)/glutathione peroxidase 4 (GPX4) axis is the key pathway to regulate ferroptosis, and cysteine depletion, inhibition of system Xc−, GSH or GPX4 can lead to ferroptosis [8, 9]. Numerous studies demonstrate that ferroptosis plays a critical role in many diseases such as, degenerative diseases, brain injury, stoke and cancers [6, 10, 11]. Notably, the high susceptibility of cancer cells to ferroptosis provides a great opportunity to treat various cancers, especially HCC [12, 13].

Ribosomal protein lateral stalk subunit p2 (RPLP2), a component of the 60S subunit of ribosomes, is located in cytoplasm [14]. Unlike the majority of ribosomal proteins which are basic, RPLP2 is acidic, and it could interact with RPLP0 and RPLP1 to form ribosomal pentameric complex (consisting of RPLP1 and RPLP2 dimers, and a RPLP0 monomer) which plays an critical role in the elongation step of protein biosynthesis [15, 16]. Many studies have demonstrated that RPLP2 is highly expressed and associated with poor prognosis in various cancer types such as, breast, colon and lung cancers, and it may involve in carcinogens by affecting the translation of specific cellular mRNAs [17–20]. However, the prognostic significance and tumorigenic effects of RPLP2 in HCC have not been studied before. In addition, it has been reported that inhibition of RPLP2 could lead to the accumulation of ROS in gynecological tumor [20], and the GSEA analysis showed that RPLP2 had a significant effect on the critical ferroptosis-related pathway “Oxidative Phosphorylation” in pediatric acute myeloid leukemia [21], indicating RPLP2 may have an effect on ferroptosis of cancer cells. However, the relationship between RPLP2 and ferroptosis is still lack of experimental verification, and the specific mechanism of RPLP2 regulating ferroptosis remains elusive.

Thus, it propels us to investigate the relationship between RPLP2 expression and its clinicopathological and prognostic significance, to explore the role of RPLP2 in ferroptosis and relevant molecular mechanism in this study, which may refine treatment effect on HCC patients.

## Materials and methods

### Gene expression analysis

The RPLP2 mRNA data across 33 cancer types and corresponding paracancer were obtained from The Cancer Genome Atlas (TCGA), and the data of normal samples were obtained from Genotype-Tissue Expression (GTEx) databases. We also utilized HCCDB which dedicated to the expression profile analyse of over 3000 HCC samples to evaluate the mRNA expression level of RPLP2. And HCC datasets GSE84402 from the GEO database was downloaded to further confirm the expression of RPLP2. The National Cancer Institute’s CPTAC dataset and Human Protein Atlas (HPA) were used to get the RPLP2 mRNA expression levels in different HCC cell lines and the location of RPLP2 in HEK293, PC3 and U2OS cell lines. R software (v 3.6.3) was used to perform statistical analysis, and visualization was achieved via the “ggplot2” (v3.3.3) package. The Wilcoxon rank-sum test was used to detect the difference between groups, and P < 0.05 was considered statistically significant.

### Correlation analysis between RPLP2 expression and clinicopathological features of HCC patients.

The clinicopathological data of the HCC patients were obtained form the TCGA-LIHC project. The differences of clinicopathological features between the high- and low-RPLP2 expression groups was analyzed via the R software (v 3.6.3). The Wilcoxon rank-sum test, chi-square test and Fisher’s precision probability test were used to detect the difference between groups. P values < 0.05 were considered statistically significant. We also used logistic regression analysis to evaluate the correlation between RPLP2 expression and the clinicopathological features of HCC patients.

### Correlation analysis between RPLP2 expression and DNA methylation level, molecular or immune subtypes in HCC

Promoter methylation level of RPLP2 in HCC tissues and corresponding normal tissues was analyzed in the UALCAN database. Additionally, we further explored the DNA methylation status in the CpG sites of the RPLP2 gene and evaluated the prognostic value of RPLP2 methylation level for HCC patients via MethSurv database (https://biit.cs.ut.ee/methsurv/). Using TISIDB database (http://cis.hku.hk/TISIDB/) which composed of many data types to evaluate the interaction between cancer and immune system, we investigated the relationship between RPLP2 expression and molecular or immune subtypes of HCC.

### Immune cell infiltration analysis

The tumor infiltration status of these 24 immune cells was evaluated by the single-sample GSEA algorithm in the “GSVA” (v1.34.0) package. The Spearman’s correlation analysis was employed to determine the correlation between the expression of RPLP2 and these immune cells. And the Wilcoxon rank-sum test was used to determine the differences of the level of immune infiltration in different RPLP2 expression groups.

### Survival prognosis analysis

Kaplan–Meier plots conducting by the “survival” (v3.2-10) package was used to investigate the relationship between RPLP2 expression and prognosis of HCC patients. And the “survminer” (v0.4.9) package was used for visualization. Using univariate and multivariate Cox regression analyses, we assessed the impact of clinical features on the prognosis of HCC patients. Prognostic variables with P < 0.1 in univariate Cox regression analysis were included in multivariate Cox regression analysis. And the “ggplot2” (v3.3.3) package was used for visualization of the forest map.

### Diagnostic value analysis

The diagnostic ROC curve and time-dependent survival ROC curve were performed using the “pROC” (v1.17.0.1) and “timeROC” (v0.4) packages to evaluate the diagnostic value of RPLP2 in HCC, and the “ggplot2” (v3.3.3) package was used for plotting. The closer the area under the curve (AUC) was to 1, the better the diagnostic accuracy was.

### Construction and validation of the nomogram

A nomogram based on RPLP2 expression and some key clinical factors was established to predict the 1-, 3-, and 5-year survival probability of the HCC patients. And the calibration plots were constructed to evaluate the prediction accuracy of the nomogram. The “RMS” (v5.1-4) package was applied to create the nomogram and calibration plots.

### Screening of DEGs

HCC patients in TCGA were divided into the high- and low-RPLP2 expression groups based on the median score of RPLP2 expression. The “DESeq2” package was used for identifying the DEGs between these two groups with thresholds of |logFC| > 1.5 and adjusted P < 0.01. Volcano plots and correlation heatmaps of the top 10 DEGs were carried out via the “ggplot2” (v3.3.3) package.

### Functional enrichment analysis

Using the “org.Hs.eg.db” (v3.10.0) package, we converted the entrez ID to the gene symbol. And functional enrichment analyses GSEA (MgDB file: c2. cp.v7.2. symbols.gmt, c5. go.v7.2. symbols.gmt) were conducted by using the “ClusterProfiler” (v3.14.3) package. |Normalized corrected ES values| (|NES|) > 1, False discovery rate (FDR) < 0.25 and nominal P-value < 0.05 was considered as significantly enriched. And the Pearson correlation analysis was performed between RPLP2 and some key ferroptosis-related genes using FerrDb V2.

### Interaction analysis

Based on DEGs (|logFC| > 1.5 and adjusted P < 0.01), PPI network was constructed and visualized via the STRING database. And the top 10 hub genes were obtained through the CytoHubba, a plugin in the Cytoscape software (version 3.7.2).

### Cell culture, transfection and chemicals

HCC cell line Hep3B, HepG2 and Huh-7 were provided by the Cancer Research Institute of Central South University, WRL68 were obtained from American Type Culture Collection (ATCC). Cells were cultured in DMEM media (Gibco, USA) with 10% fetal bovine serum (FBS), and were grown and maintained at 37 °C with 5% CO_2_ in an incubator. For subsequent assays, siRNA control and RPLP2-siRNA (5′-GGUUAUUAGUGAGCUGAAUTT-3′; 5′-GGAGUCUGAA GAGUCAGACTT-3′) (Genechem, China), using Lipofectamine® 2000 (Invitrogen) according to the manufacturer’s instructions, was transfected into cells. RPLP2 knockdown cells were obtained 72 h after transfection. Ferroptosis inducer RSL-3 (S8155) and ferroptosis inhibitor Ferrostatin-1 (S7243) were purchased from Selleck. The glutathione assay kit (S0053) was purchased form Beyotime Biotechnology.

### Western blot analysis

The collected cells were lysed in an IP lysis buffer containing a protease inhibitor cocktail. After separation by SDS-polyacrylamide gel, the total protein from cell lysis was transferred to a PVDF membrane. Membrane was incubated with primary antibodies anti-RPLP2 (823061, Zenbio), anti-GPX4 (ab125066, Abcam) and anti-β-actin (A5441, Sigma) at 4 °C overnight, followed by secondary antibodies for 2 h. The bands were visualized using the ChemiDox XRS+ image formation system.

### Immunohistochemistry

The slides were dried, dewaxed and rehydrated, followed by antigen recovery by heating the tissue with sodium citrate and blocking endogenous peroxidase by incubation with 3% hydrogen peroxide. Subsequently, the primary antibodies against RPLP2 (1:50, 823061, Zenbio), GPX4 (1:150, ab125066, Abcam), GATA3 (ZA-0661, ZSGB-BIO), CD45 (ZA-0183, ZSGB-BIO) and CD56 (ZA-0057, ZSGB-BIO) were used to stain the slides at 4 °C overnight. Then, the slides were treated with appropriate secondary antibodies at 37 °C for 30 min. Furthermore, DAB and hematoxylin were used for visual antibody staining. A pathologist Meiyuan Huang at the Department of Pathology at Zhuzhou Central Hospital verified the liver cancer biopsies.

### Cell proliferation, migration and colony formation assays

Cell viability was assessed with CCK8 kit (TP1197, Topscience) according to its instructions. Transfected cells were seeded to the wells of a 96-well plate at a concentration of 1000 cells/well, then 10 μL CCK8 solution was added to the well to incubate at 37 °C for 1 h. At last, the absorbance was measured at 450 nm by a microplate reader.

For the migration assays, 4 × 10^4^ transfected cells were seeded in upper chamber without serum, and DMEM medium with 10% FBS was added to the lower chamber. The cells on the chamber’s upper surface were removed 24 h later, and the chamber was then preserved with methanol and stained with 0.5% crystal violet. Finally, 5 random fields were taken to analyze the migration status of transfected cells.

Approximately 500 transfected cells were seeded in each well of six-well plates for the colony formation assays. After incubation for about two weeks, cells were methanol fixed and stained with 0.5% crystal violet.

### Nude mice and study approval

All animal experiments were carried out with the approval of the Xiangya School of Medicine of Central South University’s Institutional Animal Care and Use Committee. All the female nude mice at the age of four weeks were supplied by the Hunan SJA Laboratory Animal Co., Ltd. 3 × 10^6^ Hep3B cells were harvested, washed and injected subcutaneously into the back of each mouse. 27 days after injection, the mice were sacrificed and dissected, and the tumors were photographed, weighed and measured.

### Measurement of lipid ROS and total GSH

The experiments for measuring Lipid ROS and total GSH were carried out according to the instructions reported previously [12, 13]. Briefly, for the lipid ROS assay, cells were treated with RSL-3, harvested and resuspended in DMEM media with 10% FBS and C11-BODIPY (D3861, Thermo Fisher). Then samples were incubated at 37 °C and 5% CO_2_ away from light. The cells were washed twice with PBS and then resuspended in PBS. A flow cytometer (Fortessa, BD Biosciences) with fluorescein isothiocyanate (FITC) green channel and Texas red channel was used to measure the fluorescence of C11-BODIPY581 = 591.

For the detection of total GSH, after incubation with RSL-3, cells were harvested in centrifuge tubes, then incubated with 3 volumes of ice-cold 5% metaphosphoric acid, and frozen and thawed twice using liquid nitrogen and 37 °C water. After centrifugation at 12,000 rpm and 4 °C for 10 min, the total GSH was measured using the supernatant according to the protocol of the Glutathione Assay Kit.

### Drug discovery and prediction

To predict potential drugs that show certain efficacy in the RPLP2 high expression or high methylation level, we employed drug sensitivity of some drugs to analyse their treatment effect on HCC using RNAactDrug database (http://bio-bigdata.hrbmu.edu.cn/RNAactDrug/index.jsp).

## Results

### The expression of RPLP2 in HCC

**Fig. 1:**
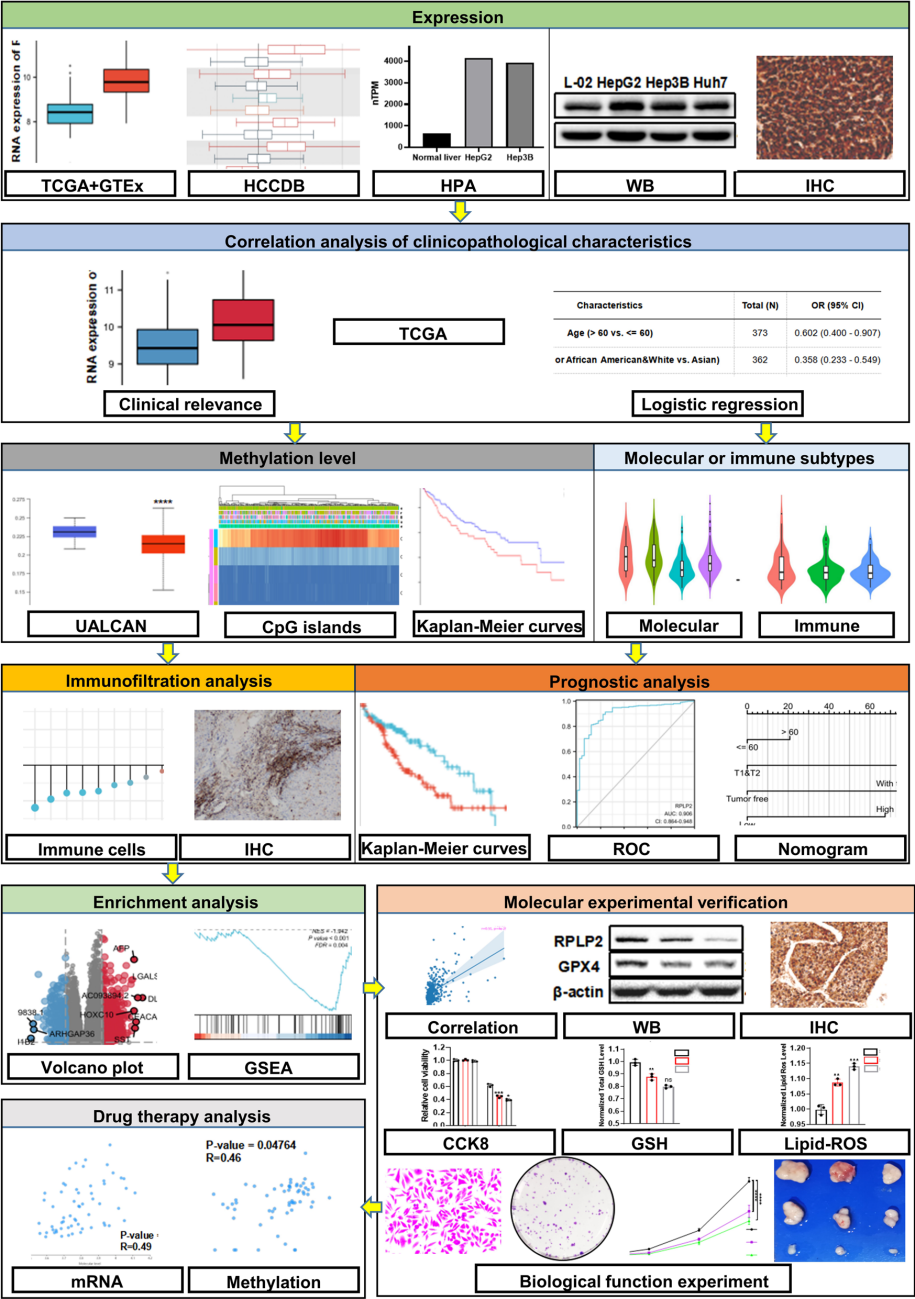
The flow chart of this study

**Fig. 2:**
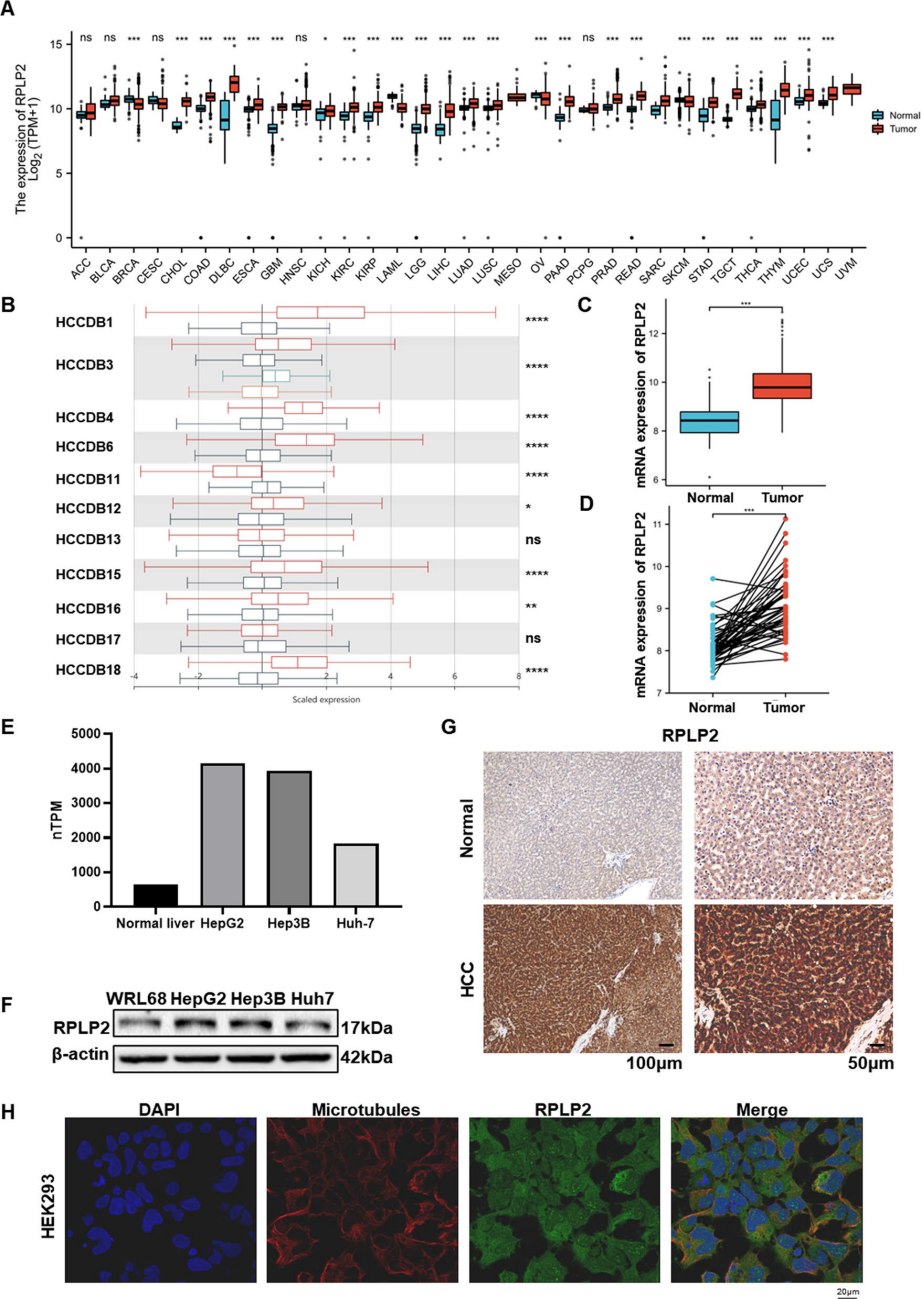
RPLP2 expression levels in HCC. **A** RPLP2 mRNA expression level in normal tissues and cancers from TCGA and GTEx databases. **B** The mRNA expression level of RPLP2 in adjacent tissues and HCC tissues from HCCDB database. **C**, **D** Unpaired (**C**) and paired (**D**) analysis of RPLP2 mRNA expression in paracancerous tissues (n = 50) and HCC tissues (n = 377) from TCGA database. **E** RPLP2 mRNA expression level in normal liver and HCC cell lines from HPA database. **F** Western blot for detecting the protein level of RPLP2 in normal live and HCC cell lines. **G** IHC test of RPLP2 protein expression in 12 pairs of clinical tissues (magnification, ×200, scale bar = 100 μm; magnification, ×400, scale bar = 50 μm). **H** The immunofluorescence staining of RPLP2 and microtubules in HEK293 cell line in HPA database. ns nonsignificant (P > 0.05), *P < 0.05, **P < 0.01, ***P < 0.001, ****P < 0.0001

The flowchart of this entire study was showed in Fig. 1. The pan-cancer analysis obtained from TCGA and GTEx showed that mRNA of RPLP2 was up-regulated in 22 types of cancer out of the 33 (Fig. 2A). The result from HCCDB database demonstrated the high expression of RPLP2 mRNA in HCC (Fig. 2B). In addition, the unpaired (Fig. 2C) and paired (Fig. 2D) sample analysis of the RPLP2 expression level from TCGA and GTEx both indicated the elevated mRNA level of RPLP2 in HCC. Moreover, compared with normal tissues, RPLP2 was also significantly higher in HCC from GSE84402 dataset (Additional file 1: Fig. S1A). To further examine the expression level of RPLP2 in different HCC cell lines, we first used the HPA dataset to find that the mRNA expression level of RPLP2 was up-regulated in HCC cell lines compared with normal liver tissue cells (Fig. 2E). Then, we detected the protein expression level of RPLP2 in normal liver cells WRL68 and several HCC cell lines, and the results showed that RPLP2 was elevated in liver cancer cells (Fig. 2F). The IHC results further proved the staining intensity of RPLP2 was greater in HCC (Fig. 2G, Additional file 1: Fig. S1B, Table S1). Additionally, in order to more intuitively and specifically detect the specific localization of RPLP2, we used the immunofluorescence results of RPLP2 from HPA to demonstrated that RPLP2 was mainly localized to the nuclear speckles and cytosol in HEK293 (Fig. 2H), PC3 and U2OS (Additional file 1: Fig. S1C, D).

### Association between RPLP2 expression and multiple clinicopathological characteristics in HCC

**Fig. 3:**
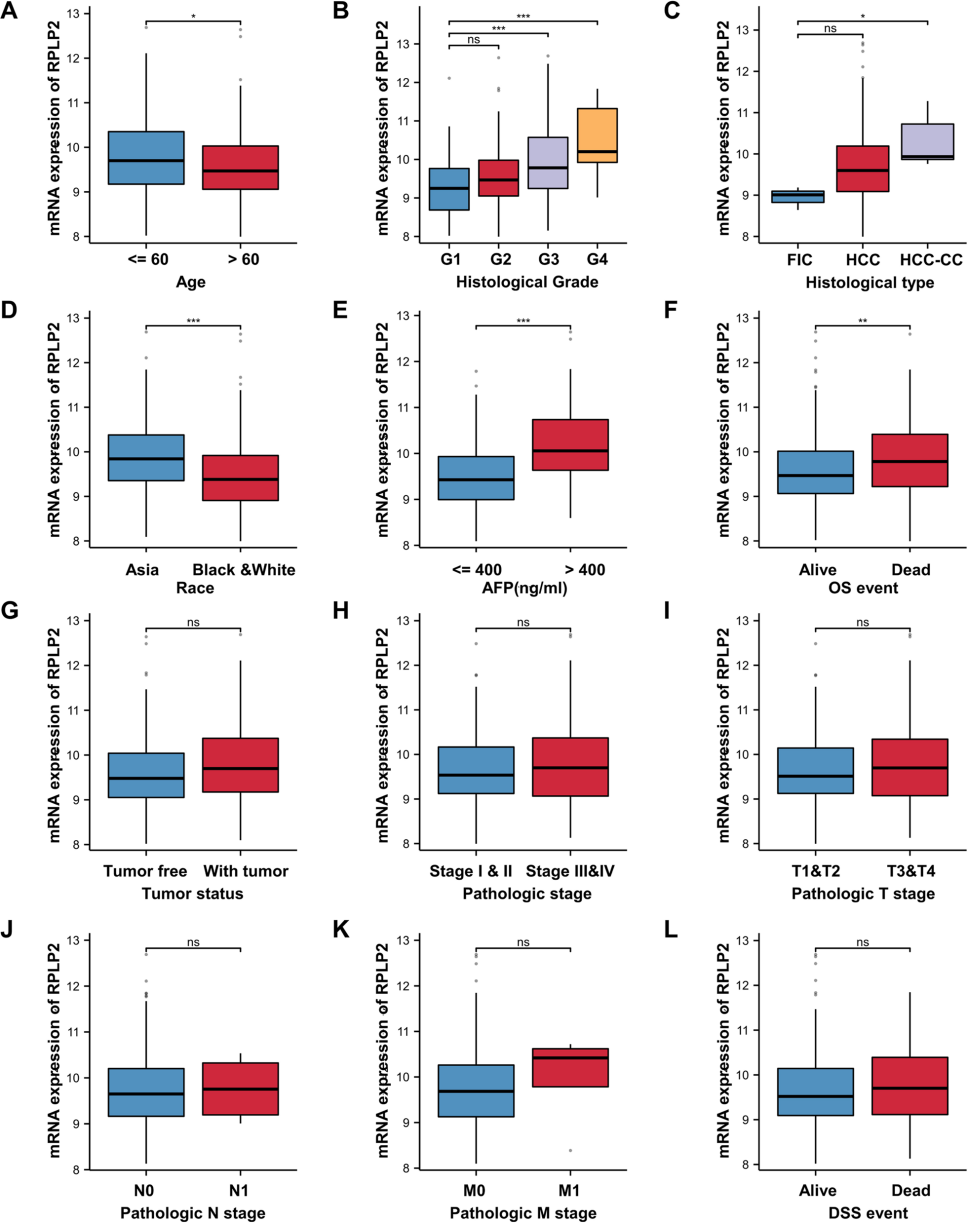
The relationship between RPLP2 expression and clinicopathological features of HCC patients. **A** Age. **B** Histological grade. **C** Histological type. **D** Race. **E** AFP levels. **F** OS. **G** Tumor status. **H** Pathologic stage. **I** T stages. **J** N stages. **K** M stages. **L** DSS. ns nonsignificant (P > 0.05), *P < 0.05, **P < 0.01, ***P < 0.001. (The data was obtained from TCGA-LIHC)

As shown in Table 1 and Fig. 3 based on the TCGA-LIHC dataset, the expression level of RPLP2 was significantly correlated with age, histological grade, histological type, race, alpha-fetoprotein (AFP) levels, overall survival (OS) and tumor status. In addition, the logistic regression analysis showed that RPLP2 expression levels significantly correlated with age [odds ratio [OR] = 0.602, 95% CI = (0.400–0.907, P = 0.015)], race [odds ratio [OR] = 0.358, 95% CI = (0.233–0.549, P < 0.001)], histological grade [odds ratio [OR] = 2.842, 95% CI = (1.829–4.417, P < 0.001)], tumor status [odds ratio [OR] = 1.651, 95% CI = (1.081–2.523, P = 0.020)], weight [odds ratio [OR] = 0.353, 95% CI = (0.228–0.546, P < 0.001)] and AFP [odds ratio [OR] = 4.094, 95% CI = (2.190–7.655, P < 0.001)] (Table 2).

**Table 1:** Clinicopathological features of high- and low-RPLP2 expression groups in HCC patients

Characteristics	Low expression of RPLP2	High expression of RPLP2	P value
n	187	187	
Age, n (%)			**0.015**
≤ 60	77 (20.6%)	100 (26.8%)	
> 60	110 (29.5%)	86 (23.1%)	
Race, n (%)			**< 0.001**
Asian	58 (16%)	102 (28.2%)	
Black or African American and White	124 (34.3%)	78 (21.5%)	
Pathologic stage, n (%)			0.201
Stage I and stage II	133 (38%)	127 (36.3%)	
Stage III and stage IV	39 (11.1%)	51 (14.6%)	
Pathologic T stage, n (%)			0.127
T1 and T2	145 (39.1%)	133 (35.8%)	
T3 and T4	40 (10.8%)	53 (14.3%)	
Pathologic N stage, n (%)			1.000
N0	117 (45.3%)	137 (53.1%)	
N1	2 (0.8%)	2 (0.8%)	
Pathologic M stage, n (%)			0.777
M0	120 (44.1%)	148 (54.4%)	
M1	1 (0.4%)	3 (1.1%)	
Histologic grade, n (%)			**< 0.001**
G1	36 (9.8%)	19 (5.1%)	
G2	102 (27.6%)	76 (20.6%)	
G3	45 (12.2%)	79 (21.4%)	
G4	1 (0.3%)	11 (3%)	
Histological type, n (%)			**0.007**
Fibrolamellar carcinoma	3 (0.8%)	0 (0%)	
Hepatocellular carcinoma	184 (49.2%)	180 (48.1%)	
Hepatocholangiocarcinoma (mixed)	0 (0%)	7 (1.9%)	
Residual tumor, n (%)			0.674
R0	165 (47.8%)	162 (47%)	
R1 and R2	10 (2.9%)	8 (2.3%)	
Tumor status, n (%)			**0.020**
Tumor free	111 (31.3%)	91 (25.6%)	
With tumor	65 (18.3%)	88 (24.8%)	
AFP (ng/mL), n (%)			**< 0.001**
≤ 400	123 (43.9%)	92 (32.9%)	
> 400	16 (5.7%)	49 (17.5%)	
Adjacent hepatic tissue inflammation, n (%)			0.322
None	71 (30%)	47 (19.8%)	
Mild	51 (21.5%)	50 (21.1%)	
Severe	11 (4.6%)	7 (3%)	
Vascular invasion, n (%)			0.180
No	111 (34.9%)	97 (30.5%)	
Yes	50 (15.7%)	60 (18.9%)	
Albumin(g/dl), n (%)			0.392
< 3.5	39 (13%)	30 (10%)	
≥ 3.5	117 (39%)	114 (38%)	
OS event, n (%)			**0.002**
Alive	136 (36.4%)	108 (28.9%)	
Dead	51 (13.6%)	79 (21.1%)	
DSS event, n (%)			0.099
No	150 (41%)	137 (37.4%)	
Yes	33 (9%)	46 (12.6%)	

Bold values represent statistically significant *p*–value

**Table 2:** Logistic regression analysis of the relationship between RPLP2 expression levels and clinicopathological characteristics in HCC patients

Characteristics	Total (N)	OR (95% CI)	P value
Age (> 60 vs. ≤ 60)	373	0.602 (0.400–0.907)	**0.015**
Race (Black or African American and White vs. Asian)	362	0.358 (0.233–0.549)	**< 0.001**
Pathologic stage (Stage III and Stage IV vs. Stage I and Stage II)	350	1.369 (0.845–2.219)	0.202
Pathologic T stage (T3 and T4 vs. T1 and T2)	371	1.445 (0.900–2.319)	0.128
Pathologic N stage (N1 vs. N0)	258	0.854 (0.118–6.157)	0.876
Pathologic M stage (M1 vs. M0)	272	2.432 (0.250–23.683)	0.444
Histologic grade (G3 and G4 vs. G1 and G2)	369	2.842 (1.829–4.417)	**< 0.001**
Residual tumor (R1 and R2 vs. R0)	345	0.815 (0.314–2.117)	0.674
Tumor status (with tumor vs. tumor free)	355	1.651 (1.081–2.523)	**0.020**
Weight (> 70 vs. ≤ 70)	346	0.353 (0.228–0.546)	**< 0.001**
Adjacent hepatic tissue inflammation (mild and severe vs. none)	237	1.389 (0.830–2.324)	0.211
Vascular invasion (yes vs. no)	318	1.373 (0.864–2.183)	0.180
AFP (ng/mL) (> 400 vs. ≤ 400)	280	4.094 (2.190–7.655)	**< 0.001**

Bold values represent statistically significant *p*–value

### Correlation between RPLP2 expression with methylation level, molecular or immune subtypes in HCC

**Fig. 4:**
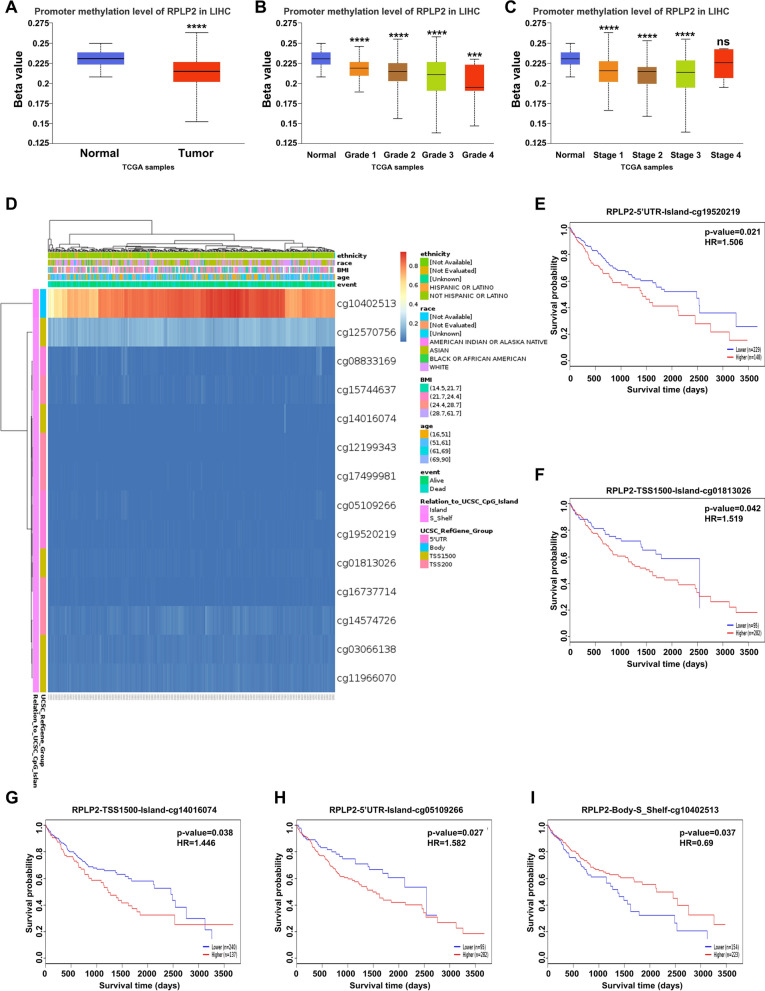
DNA methylation level of RPLP2 and its relationship with the prognosis of HCC patients. **A** The promoter methylation features of RPLP2 in HCC obtained from UALCAN database (n of normal = 165, n of tumor = 165). **B**, **C** The promoter methylation level of RPLP2 was analyzed by the tumor grade (**B**) and the main pathological stages (**C**) of HCC via UALCAN database. **D** Correlation between RPLP2 expression and methylation level in HCC obtained from MethSurv database. **E**–**I** Kaplan–Meier survival curves showing the effect of methylation levels in the CpG sites of RPLP2 on the prognosis of HCC patients obtained form MethSurv database. ns nonsignificant (P > 0.05), ***P < 0.001, ****P < 0.0001

For elucidating the potential mechanism of RPLP2 overexpression in HCC tissues, we first used UALCAN database to explore the relationship between RPLP2 expression and DNA methylation levels of the promoter. The results demonstrated that HCC tissues showed an obviously lower level of promoter methylation than normal liver tissues (Fig. 4A), and the expression level of RPLP2 was negatively correlated to the tumor grades and individual cancer stages separately (Fig. 4B, C). Furthermore, we explored the specific methylation status of different methylation sites of RPLP2 and its correlation with the prognosis of HCC patients via using MethSurv tool. The results indicated that most of methylation sites in the DNA sequences of RPLP2 were hypomethylated in HCC (Fig. 4D), and methylation level of five CpG islands were associated with patient outcomes. Specifically, elevated methylation levels of RPLP2 in four islands including cg19520219, cg01813026, cg14016074 and cg05109266 were correlated with poor prognosis (Fig. 4E–I). In addition, we further analyzed the correlation between RPLP2 expression and molecular or immune subtypes in HCC from the TISIDB database. The analysis results indicated that there was no obvious difference shown in different molecular subtypes (Additional file 1: Fig. S2A), but for immune subtypes, RPLP2 expression was significantly different in HCC (Additional file 1: Fig. S2B).

### Correlation between RPLP2 expression and the infiltration of multiple immune cell types in HCC

**Fig. 5:**
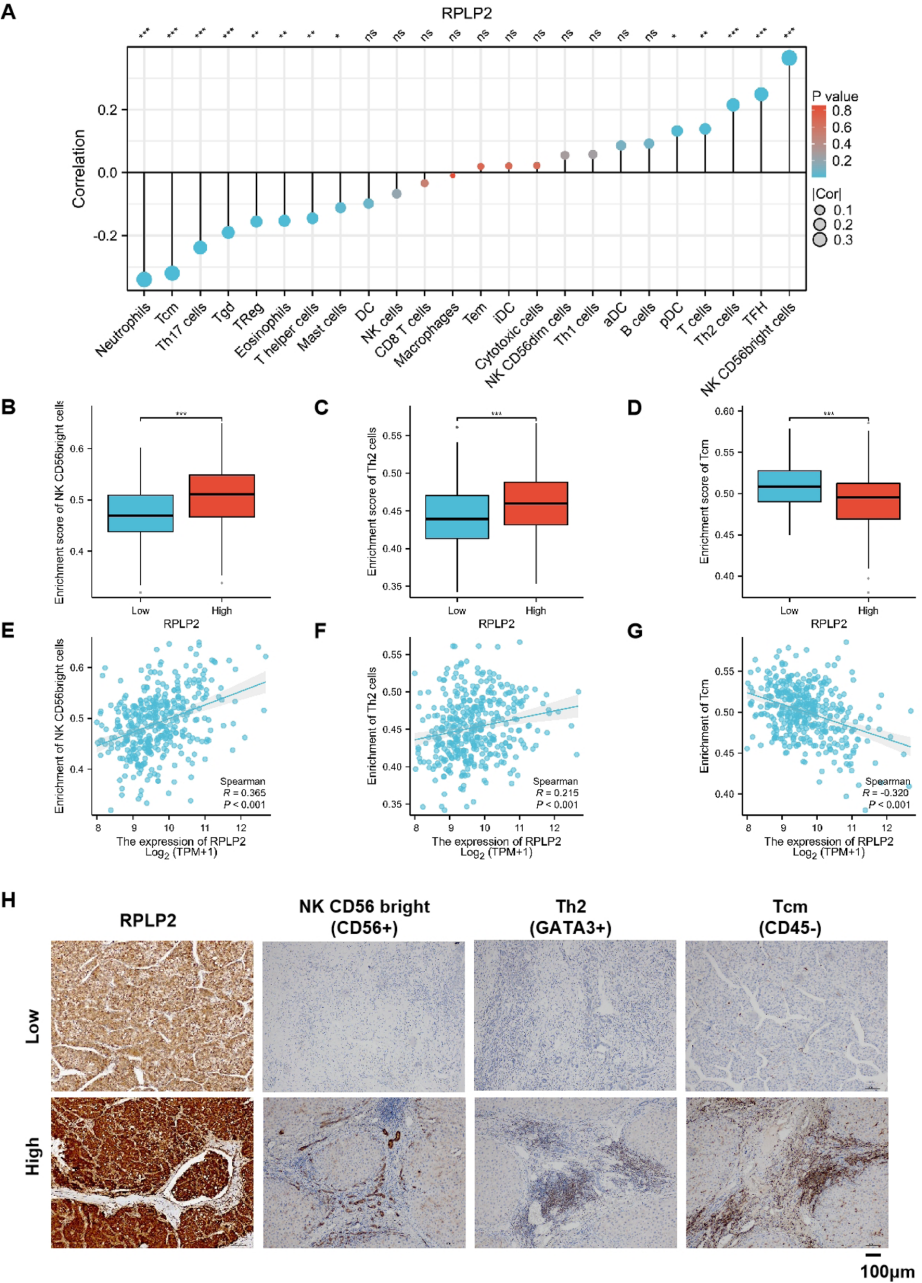
The correlation between RPLP2 expression and immune cell infiltration in HCC. **A** Spearman’s correlation analysis between RPLP2 expression and relative abundance of 24 types of immune infiltrating cells. **B**–**D** The immune infiltration levels of NK CD56 bright (**B**), Th2 (**C**) and Tcm (**D**). **E**–**G** The relationship between RPLP2 expression and NK CD56 bright (**E**), Th2 (**F**) and Tcm (**G**). (The data was obtained from TCGA-LIHC). **H** IHC test verified the infiltration level of immune cells in HCC with high or low RPLP2 expression (magnification, ×200, scale bar = 100 μm). ns nonsignificant (P > 0.05), *P < 0.05, **P < 0.01, ***P < 0.001

ssGSEA was used to evaluate the infiltration status of 24 kinds of immune cells, and the association between RPLP2 expression and immune cell infiltration was estimated by the Spearman’s correlation analysis (Fig. 5A). The analysis result showed that RPLP2 significantly positively correlated with NK CD56 bright and Th2, and negatively correlated with Tcm (Fig. 5B–D). In addition, the enrichment scores of NK CD56 bright, Th2 and Tcm were consistent with the Spearman’s analysis results (Fig. 5E–G). Moreover, the results of IHC further proved that high RPLP2 expression was associated with more NK CD56 bright and Th2 cell infiltration and less infiltration of Tcm cells in HCC (Fig. 5H).

### Potential prognostic and diagnostic value of RPLP2 in HCC

**Fig. 6:**
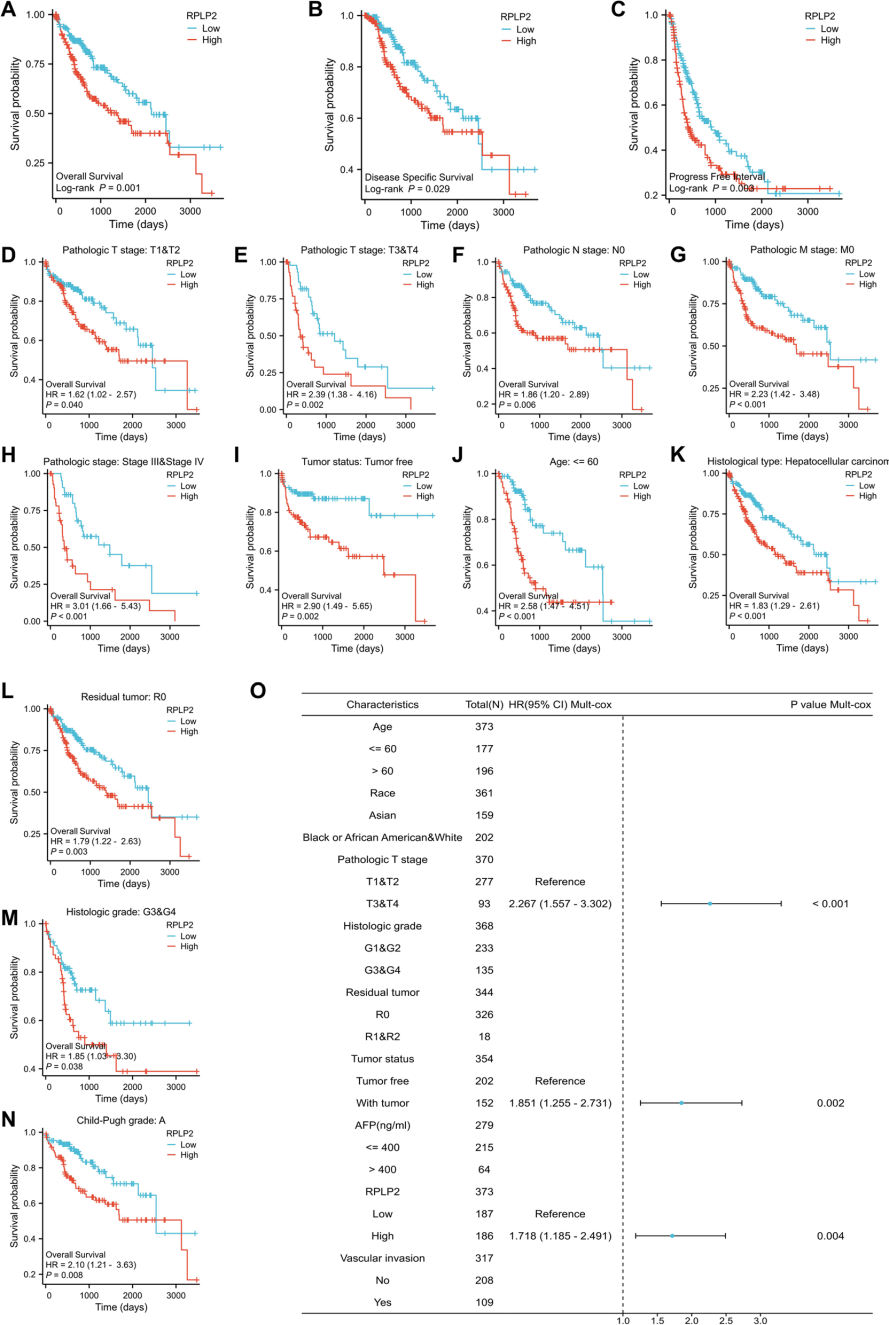
Survival analysis of RPLP2 in HCC. **A**–**C** Kaplan–Meier curves for patient’s OS (**A**), DSS (**B**) or PFI (**C**) classified by different expression level of RPLP2 in HCC (n of low = 187, n of high = 186). **D**–**N** Kaplan–Meier curves indicating the OS prognostic value of RPLP2 expression in different HCC subgroups including, T1 and T2 (**D**) (n of low = 139, n of high = 138), T3 and T4 (**E**) (n of low = 46, n of high = 47), N0 (**F**) (n of low = 127, n of high = 127), M0 (**G**) (n of low = 134, n of high = 134), Stage III and IV (**H**) (n of low = 45, n of high = 45), tumor free (**I**) (n of low = 101, n of high = 101), age ≤ 60 (**J**) (n of low = 88, n of high = 89), hepatocellular carcinoma (**K**) (n of low = 182, n of high = 181), R0 (L) (n of low = 163, n of high = 163), G3 and G4 (**M**) (n of low = 68, n of high = 67) and Child–Pugh grade A (**N**) (n of low = 109, n of high = 109). **O** Forest plots showing the potential prognostic indicators for OS. (The data was obtained from TCGA-LIHC)

Kaplan–Meier method was carried out to analyse the relationship between RPLP2 expression and the prognosis of HCC patients. The survival curve showed that compared with low RPLP2 expression group, HCC patients with high RPLP2 level exhibited worse prognosis of OS, DSS and PFI (Fig. 6A–C). Then we further explored the effect of RPLP2 on prognosis in different subgroups of HCC patients. And the results indicated that the high level of RPLP2 predicted unfavorable OS in various subgroups including T1 and T2, T3 and T4, N0, M0, Stage III and IV, tumor free, age ≤ 60, hepatocellular carcinoma, R0, G3 and G4 and Child–Pugh grade A (Fig. 6D–N). For DSS, RPLP2 played a risk role in several subgroups, such as Stage III and IV, age ≤ 60, hepatocellular carcinoma and R0 (Additional file 1: Fig. S3A–D). And the prognosis of PFI was notably poor in many subgroups including N0, MO, with tumor, age ≤ 60, hepatocellular carcinoma, R0 and G1 and G2 (Additional file 1: Fig. S3E–K). In addition, we used univariate and multivariate cox regression analyses to figure out potential prognostic indicators (Additional file 1: Tables S2–S4). The multivariate cox regression analysis demonstrated that the pathological T stage was an independent risk factor of OS and DSS, the tumor status and the expression level of RPLP2 were valuable prognostic predictors of OS and PFI, and the vascular invasion exhibited a great value in clinical predicting PFI (Fig. 6O, Additional file 1: Fig. S4A, B).

**Fig. 7:**
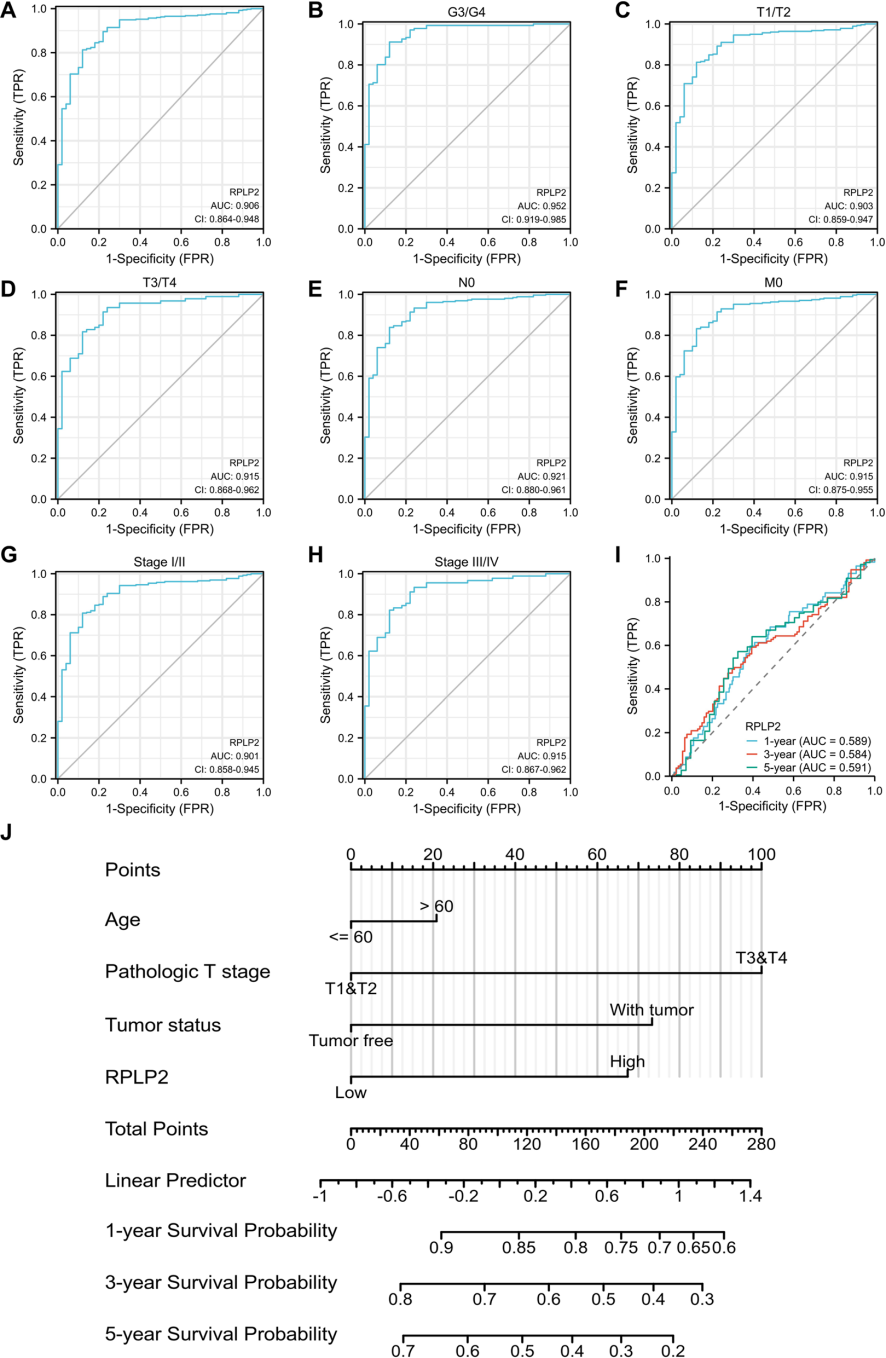
The nomogram with RPLP2 shows excellent performance in the diagnosis and prognosis of HCC. **A** ROC analysis of RPLP2 in HCC. **B**–**H** Diagnostic value of RPLP2 mRNA level in different HCC subgroups including, G3 and G4 (**B**), T1 and T2 (**C**), T3 and T4 (**D**), N0 (**E**), M0 (**F**), Stage I and II (**G**) and Stage III and IV (**H**). **I** Time-dependent survival ROC analysis for predicting the probability of HCC patients with 1-, 3- and 5-year survival. **J** Nomogram for predicting the 1-, 3- and 5-year overall survival rates of HCC patients. (The data was obtained from TCGA-LIHC)

We plotted the receiver operating curve (ROC) to investigate the diagnostic value of RPLP2 in HCC. And the ROC curve analysis demonstrated that RPLP2 had great performance (AUC = 0.906) in distinguishing HCC tumor from normal control (Fig. 7A). Then, we evaluated the diagnostic value of RPLP2 expression in different subgroup of HCC patients. Specifically, RPLP2 exhibited excellent diagnostic value (AUC > 0.90) in many subgroups including.

G3 and G4, T1 and T2, T3 and T4, N0, M0, Stage I and II and Stage III and IV (Fig. 7B–H). In addition, the result of time-dependent ROC curve showed that RPLP2 had certain prediction accuracy (AUC = 0.589, 0.584 and 0.591) for 1-, 3-, and 5-year survival rates of HCC patients (Fig. 7I). At last, we established a nomogram combining RPLP2 expression and some critical clinical features which exhibited significantly high value in predicting the 1-, 3-, and 5-year survival probability of the HCC patients (Fig. 7J, Additional file 1: Fig. S5A–C).

### DEGs between high- and low-RPLP2 expressing HCC patients and PPI network analysis

Using absolute log-fold change > 1.5 and P < 0.01 as the threshold parameters, we identified 1141 differentially expressed genes (777 up-regulated and 364 down-regulated) between high- and low-RPLP2 expressing HCC groups (Additional file 1: Fig. S6A). The top ten significant DEGs (including LGALS14, DLK1, AC093894.2, CYP11B2, CEACAM7, SST, AL109838.1, AFP, HOXC10 and ARHGAP36) were shown the single gene co-expression heat map (Additional file 1: Fig. S6B). In order to explore the interactions between all RPLP2-related DEGs, we carried out the online STRING tool to construct a PPI network (Additional file 1: Fig. S6C), and further used cytoscope to figure out the top 10 hub genes which were CHGA, ISL1, AFP, ASCL1, CALB2, KRT19, NKX2-2, SST, EPCAM and ESR1 (Additional file 1: Fig. S6D).

### Knockdown of RPLP2 promotes ferroptosis

**Fig. 8:**
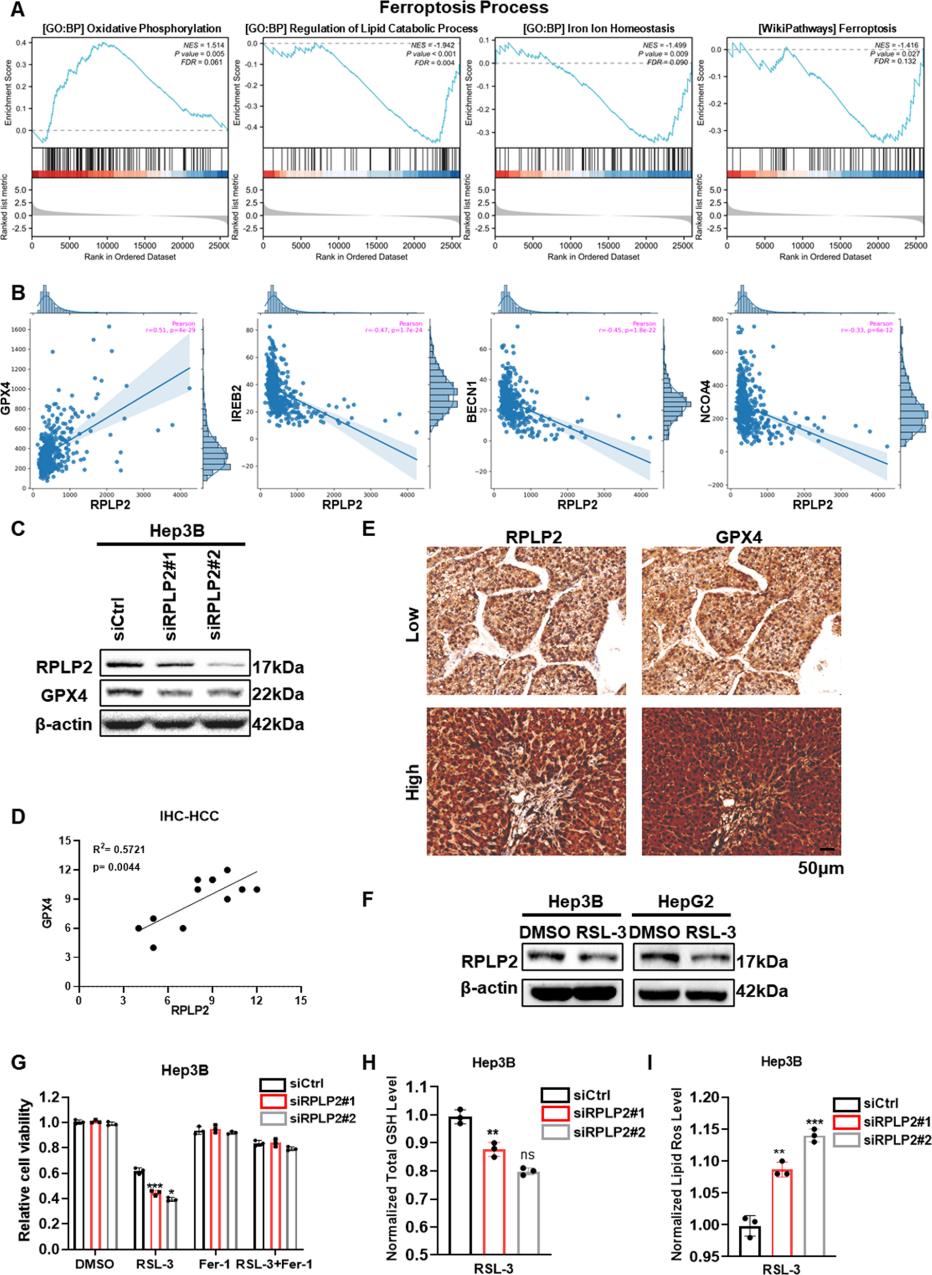
RPLP2 silencing promotes ferroptosis of HCC cells. **A** Gene set enrichment plots of “Oxidative Phosphorylation”, “Regulation of Lipid Catabolic Process”, “Iron Ion Homeostasis” and “WP Ferroptosis” from GSEA of RPLP2-related DEGs. (The data was obtained from TCGA-LIHC). **B** The correlation between RPLP2 expression and key ferroptosis gene regulators GPX4, IREB2, BECN1 and NCOA4 via FerrDb V2. **C** The expression levels of RPLP2 and GPX4 in Hep3B cells with RPLP2 knockdown were detected using western blot. **D** The correlation between RPLP2 and GPX4 protein level was analyzed in 12 clinical HCC tissues. **E** IHC analysis of the relationship between RPLP2 and GPX4 expression in 12 clinical HCC tissues. **F** The expression level of RPLP2 in Hep3B cells and HepG2 cells treated with ferroptosis inducer RSL-3 were detected via western blot. **G** CCK8 assays detected the responses of Hep3B cells knockdown of RPLP2 to Fer-1 and RSL-3. (n = 3 independent experiments). **H**, **I** The levels of total GSH (H) and lipid ROS (I) in Hep3B cells knockdown of RPLP2 were detected. (n = 3 independent experiments). ns nonsignificant (P > 0.05), *P < 0.05, **P < 0.01, ***P < 0.001

Considering the accumulation of ROS caused by the inhibition of RPLP2 in gynecologic tumors [20], and the significant effect of RPLP2 on ferroptosis-related pathway showed by GSEA in AML [21], we further investigated whether RPLP2 had an effect on ferroptosis of HCC cells. First, we used Gene set enrichment analysis (GSEA) to reveal that several critical ferroptosis-related pathways including “Oxidative Phosphorylation”, “Regulation of Lipid Catabolic Process”, “Iron Ion Homeostasis” and “WP Ferroptosis” exhibited significant enrichment differences between high and low RPLP2 expression groups (Fig. 8A). In addition, the correlation analysis between RPLP2 and key ferroptosis gene regulators in TCGA HCC samples from FerrDb showed that RPLP2 positively correlated with ferroptosis suppressor gene GPX4, and negatively associated with ferroptosis driver genes IREB2, BECN1 and NCOA4 (Fig. 8B). Furthermore, the result of western blot demonstrated that GPX4 was decreased by the knockdown of RPLP2 (Fig. 8C), and IHC assay also proved that the staining intensity of RPLP2 was positively correlated with GPX4 in HCC (Fig. 8D, E). Next, we treated Hep3B and HepG2 cells with ferroptosis inducer RSL-3 which could activate oxidative stress pathways, and we found that compared with the DMSO treated group, Hep3B and HepG2 cells treated with RSL-3 had a decreased expression level of RPLP2 (Fig. 8F). Then, we treated Hep3B cells with RSL-3, and the CCK8 analysis indicated that RPLP2 knockdown promoted the ferroptosis of Hep3B cells (Fig. 8G). Moreover, the detection of GSH and lipid ROS (surrogate markers for ferroptosis) in Hep3B cells treated with RSL-3 showed that RPLP2 knockdown could lead to the decrease of GSH (Fig. 8H) and increase of lipid ROS (Fig. 8I). Notably, we further explored whether RPLP2 upregulation was linked to ferroptosis in seven other cancers where RPLP2 was most significantly upregulated, and the results indicated that RPLP2 also showed significant correlation with GPX4 in five cancer types including GBM, LGG, PAAD, TGCT and THYM (Additional file 1: Fig. S7A). And GSEA results indicated that RPLP2 expression significantly alter the enrichment of several ferroptosis-related pathways in these five cancer types, especially in THYM (Additional file 1: Fig. S7B–D).

### RPLP2 knockdown inhibits tumor growth

**Fig. 9:**
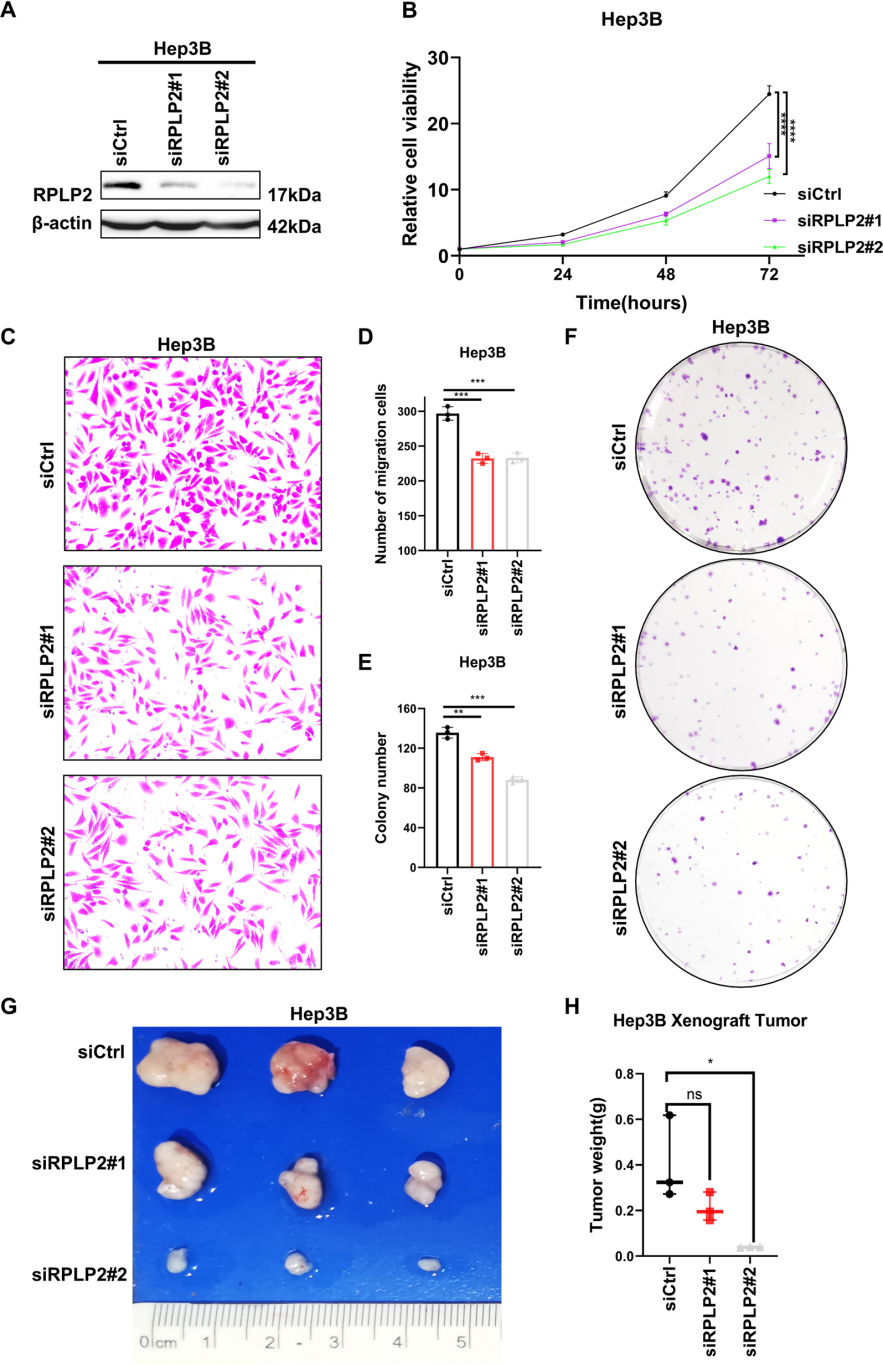
Knockdown of RPLP2 inhibits tumor growth of HCC. **A** Western blot detecting the transfection efficiency of siRPLP2#1 and siRPLP2#2 in the Hep3B cell. **B** CCK8 assays was applied to detect the effect of RPLP2 knockdown on the cell proliferation of Hep3B cells. (n = 3 independent experiments). **C**, **D** The transwell assay of Hep3B cells with RPLP2 knockdown. (n = 3 independent experiments). **E**, **F** The effect of RPLP2 knockdown on the colony formation of Hep3B cells was evaluated by colony formation assay. (n = 3 independent experiments). **G**, **H** The nude mice xenograft model was established to explore the effect of RPLP2 knockdown on tumor volume (**G**) and weight (**H**). (n = 3 mice per group). ns nonsignificant (P > 0.05), *P < 0.05, **P < 0.01, ***P < 0.001, ****P < 0.0001

To explore the role of RPLP2 in HCC, we first selected Hep3B cell line which had a high RPLP2 expression level for functional analysis in vitro. The results of CCK8, transwell and colony formation assays indicated that RPLP2 silencing significantly reduced the cell proliferation, cell migration and colony formation ability of Hep3B cells (Fig. 9A–F). Then we further confirm the tumorigenic function of RPLP2 on HCC in vivo. After transplanting RPLP2 knockdown Hep3B cells subcutaneously into nude mice, we found that tumor size and weight were obviously decreased upon the silencing of RPLP2 (Fig. 9G, H). Taken together, these findings suggested that RPLP2 promoted HCC growth and migration.

### Potential therapeutic drugs targeting RPLP2 for treatment

**Fig. 10:**
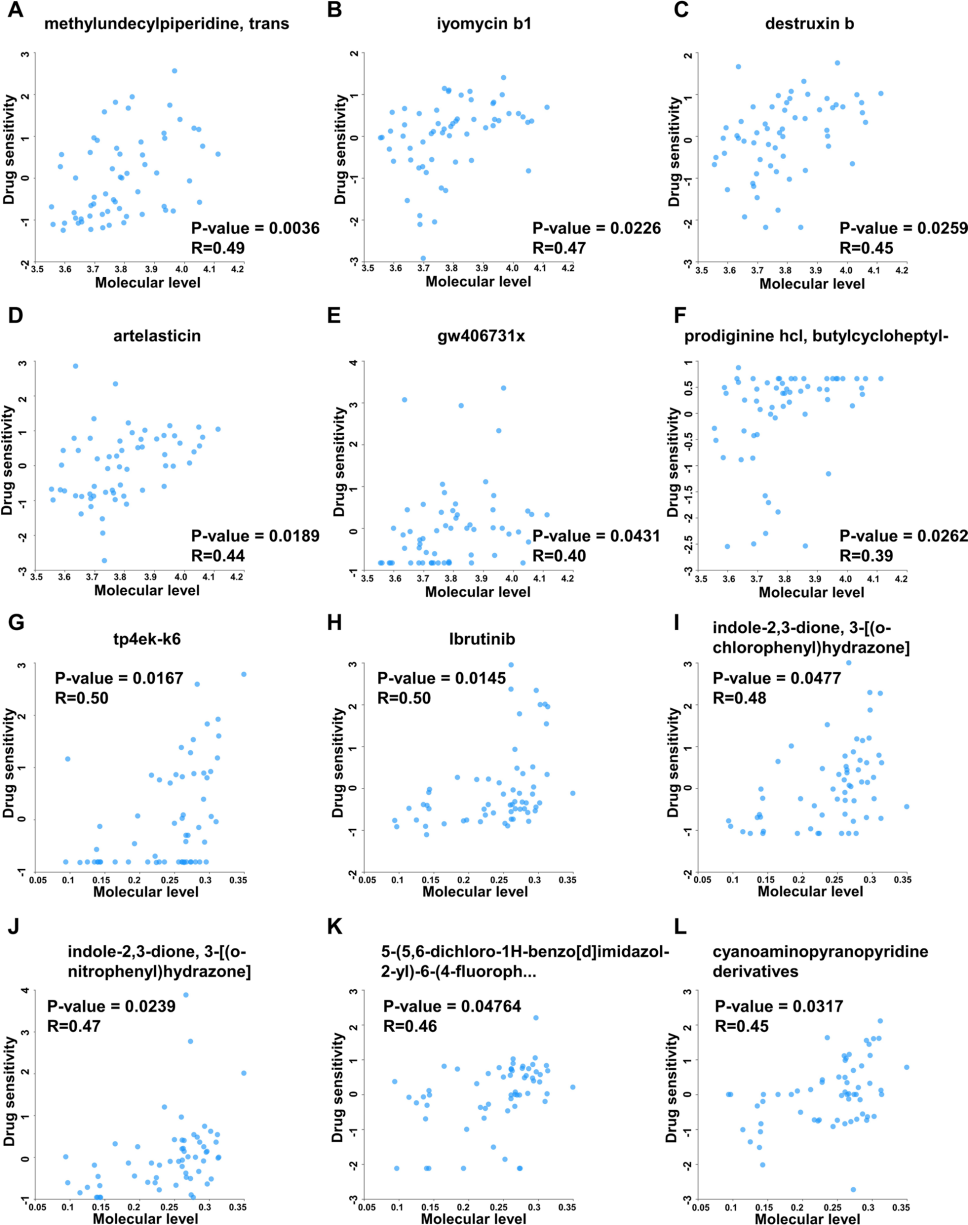
Predicting potential drugs targeting RPLP2 for treatment. **A**–**F** Correlation analysis between drug sensitivity and mRNA expression of RPLP2 based on RNAactDrug database. **G**–**L** Correlation analysis between drug sensitivity and methylation level of RPLP2 based on RNAactDrug database

Considering the critical role of RPLP2 in promoting liver cancer, it’s necessary to search for drugs specifically targeting RPLP2 with high sensitivity. We used the RNAactDrug database to analyse the correlation between the drug sensitivity and mRNA expression or methylation level of RPLP2. The results showed that the drug sensitivity of methylundecylpiperidine, trans, iyomycin b1, destruxin b, artelasticin, gw406731x and prodiginine hcl, butylcycloheptyl-increased with elevated mRNA expression of RPLP2 (Fig. 10A–F). In addition, we found that patients with high RPLP2 methylation level had great sensitivity for tp4ek-k6, Ibrutinib, indole-2,3-dione, 3-[(*o*-chlorophenyl)hydrazone], indole-2,3-dione, 3-[(*o*-nitrophenyl)hydrazone], 5-(5,6-dichloro-1*H*-benzo[d]imidazol-2-yl)-6-(4-fluoroph… and cyanoaminopyranopyridine derivatives (Fig. 10G–L).

## Discussion

Metastasis at the advanced stage is the leading cause of HCC-related mortality [22]. Despite landmark progress in HCC diagnosis in recent years, the prognosis of HCC patients remains poor [23]. Thus, identification of novel biomarkers is critical to enhance individualized therapies. The ribosomal stalk, consisting of acidic ribosomal proteins RPLP0, RPLP1, and RPLP2, played an essential role in promoting translation subsets of cellular mRNAs, and closely linked to several pathological conditions, including autoimmune diseases and human malignancies [17, 24]. Studies indicated that RPLP1 was up-regulated and significantly associated with poor prognosis in HCC, and overexpression of RPLP1 promoted the proliferation, migration and invasion of Hep3B cells [25]. In addition, RPLP0 was also proved to be a potential novel biomarker for the treatment, diagnosis, and prognosis of HCC [26]. Many studies indicate that RPLP2 has been linked closely to the tumorigenesis, progression and malignant behavior of various cancers such as breast, lung and ovarian cancers [19, 20]. However, the specific role of RPLP2 in HCC has not been studied before. Here, we systematically explored the expression profile, clinicopathological, prognostic and diagnostic significance, DNA methylation level, immunomodulatory effects, the effect on ferroptosis and potential targeted therapy strategies of RPLP2 in HCC.

As a candidate oncogene, RPLP2 is overexpressed and closely associated with occurrence and progression of various cancers including but not limited to breast, ovarian, colon, lung cancer and AML [18–21], while there is no reports about the effect of RPLP2 on HCC. At present study, we first analyzed the expression level of RPLP2 in HCC. Using TCGA+GTEx, HCCDB, GEO and HPA, we found that RPLP2 mRNA was highly expressed in HCC compared with normal liver tissue, and the results of IHC and western blot further demonstrated it on the protein level. In addition, RPLP2 expression was correlated with clinical characteristics including age, histological grade, histological type, race, AFP levels, OS and tumor status, and logistic regression analysis showed that RPLP2 expression was significantly associated with age, race, histological grade, tumor status, weight and AFP levels. Moreover, Kaplan–Meier test demonstrated that HCC patients with elevated RPLP2 expression had undesirable OS, DSS and PFI, which was also exhibited in many clinical categories, according to subgroup survival analyses. The multivariate cox regression analysis indicated high RPLP2 expression was an independent risk factor for OS and PFI in HCC. What’ more, the ROC curve analysis showed that RPLP2 had a great diagnostic performance for HCC.

DNA methylation, a common epigenetic mechanism, generally silencing gene expression by altering chromatin structure and DNA stability, plays a significant role in tumorigenesis [27, 28]. However, studies on the methylation level of RPLP2 in cancers are rare. To our knowledge, it has only been reported that RPLP2 is hypermethylated in CRC [29]. Here, we found that the high expression of RPLP2 in HCC may be linked to its promoter hypomethylation, and the survival analysis indicated that hypomethylated level of RPLP2 correlated significantly with the prognosis of HCC patients. Additionally, the meaningful correlation between RPLP2 expression and different immune subtypes, suggesting us to explore the function of RPLP2 in cancer deeply by targeting specific immune subtypes.

Tumor immune microenvironment (TIME), mainly composed of immune cells, plays a significant role in the occurrence and progression of cancer, which affects clinical prognosis and immunotherapy effect greatly [30, 31]. Previous study indicated that recombinant RPLP2 with Toll-like receptor 4 (TLR4) could induce the maturation and activation of DCs, and pulse tumor-specific antigen, to induce the activation of tumor-specific CD8IFN-γ T cells followed by tumor clearance [15]. In our study, we found that RPLP2 positively correlated with NK CD56 bright and Th2 cells, and strongly negatively correlated with Tcm cells, which was also verified by IHC results. NK CD56 bright cells have been proved to have high infiltration levels and promote tumorigenesis in breast, colorectal and lung cancers [32–34]. And Th2 cells was reported to be associated with immunosuppression and poor prognosis in various cancer types [35]. According to a recent study, strategies aimed at inducing Tcm in TLSs/E-TLSs combined with immune checkpoint inhibitors, might represent promising avenues for cancer treatment [36]. Conclusively, these results indicated that the elevated level of RPLP2 may affect the tumorigenesis of HCC by regulating the immune cell infiltration levels.

Ferroptosis, a newly defined form of iron-dependent non-apoptotic cell death caused by lipid peroxidation [37]. Triggering ferroptosis is considered to be a effective strategy on suppressing tumor progression [38, 39]. As a crucial regulator of ferroptosis, GPX4 plays a significant role of phospholipid hydroperoxides, and inactivation of GPX4 could accelerate the progression ferroptosis [40, 41]. Studies have demonstrated that the silencing of RPLP2 could lead to the accumulation of ROS in gynecological tumor [16], and RPLP2 has an obvious effect on the critical ferroptosis-related pathway “Oxidative Phosphorylation” in AML showed by GSEA analysis [21]. However, the specific role of RPLP2 in HCC has not been studied, and its molecular mechanism remains unclear. In this study, we first found that RPLP2 played a critical role in ferroptosis-related pathways including “Oxidative Phosphorylation”, “Regulation of Lipid Catabolic Process”, “Iron Ion Homeostasis” and “WP Ferroptosis” using GSEA. Then the correlation analysis showed that RPLP2 significantly positively linked with GPX4 in HCC, which was further confirmed by western blot and IHC. Additionally, the CCK8 analysis indicated that inhibition of RPLP2 accelerated ferroptosis of HCC cells. Moreover, RPLP2 knockdown lead to the decrease of GSH and increase of lipid ROS. What’s more, the correlation analysis and GSEA results in other cancers where RPLP2 was significantly upregulated indicated that PRLP2 may play an important role in inhibiting ferroptosis of cancer cells through GPX4 in GBM, LGG, PAAD, TGCT and especially THYM, and further experimental verification was needed. Conclusively, these results demonstrated that RPLP2 is an important regulator of ferroptosis, which could be a potential target to eradicate HCC cells.

Silencing of RPLP2 may result in inhibition of proliferation of several cancer types of cells [20, 42]. However, the concrete effect of RPLP2 expression on the malignant phenotype of HCC remains unclear. Here, we discovered RPLP2 knockdown significantly suppressed the proliferation, migration and colony formation of HCC cells via in vitro experiments, and xenograft nude mice model further proved that tumor growth was inhibited after knockdown of RPLP2. Combined with the role of RPLP2 in ferroptosis of HCC cells, these results suggest that RPLP2 could promote cell proliferation and tumor growth of HCC by inhibiting ferroptosis.

The key role of RPLP2 in HCC development prompts us to explore highly sensitive drugs that specifically target RPLP2. Using the RNAactDrug database [43], we noticed that some commonly used anticancer drugs such as, methylundecylpiperidine, trans, iyomycin b1, and destruxin b showed high sensitivity in patients with high RPLP2 mRNA levels. In addition, patients with elevated methylation level of RPLP2 were significantly sensitive to tp4ek-k6, Ibrutinib and indole-2,3-dione, 3-[(o-chlorophenyl)hydrazone]. These findings suggested that RPLP2 may have the potential to be targeted to bring new advances in HCC therapy after well-established preclinical and clinical trials.

**Fig. 11:**
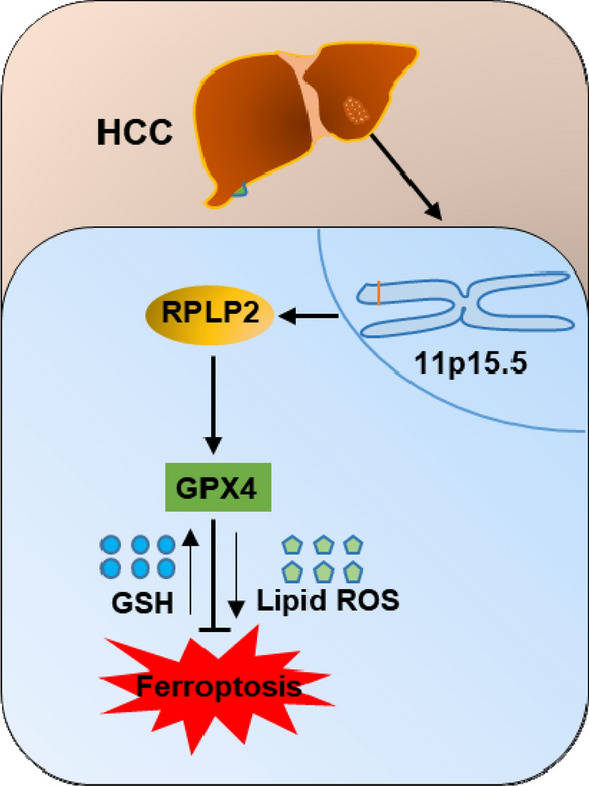
RPLP2 positively correlates with GPX4 and inhibits ferroptosis of HCC cells

Generally, our present study revealed that high expression of RPLP2 is an independent adverse prognostic factor in HCC, and is significantly correlated with aggressive clinical characteristics, tumor growth and inhibition of ferroptosis. Mechanically, RPLP2 positively links to anti-ferroptosis protein GPX4, which then protects cells from ferroptosis to promote the progression of HCC (Fig. 11). Our findings suggest that targeting RPLP2 to promote the ferroptosis of HCC cells could be a potential therapeutic strategy for HCC patients. However, the concrete regulatory mechanism of RPLP2 on GPX4 has not been explored in this study. And although we found that RPLP2 is strongly associated with immune infiltration, the underlying molecular mechanism and signaling pathways are not further investigated.

### Supplementary Information

**Additional file 1: Figure S1.** The expression level of RPLP2 in HCC and its localization in cancer. (A) RPLP2 mRNA expression level in peritumoral liver tissues and HCC tissues in GSE84402 database. (B) IHC test of RPLP2 protein expression in HCC. (C, D) Immunofluorescence assay of RPLP2 and microtubules in PC3 (C) and U2OS (D) cell lines from HPA database. *P < 0.05, ****P < 0.0001. **Figure S2.** Correlations between RPLP2 expression and molecular/immune subtypes in HCC. (A) Correlations between RPLP2 expression and molecular subtypes in HCC obtained from TISIDB database. (B) Correlations between RPLP2 expression and immune subtypes in HCC obtained from TISIDB database. **Figure S3.** DSS and PFI survival curve in different subgroups between high- and low-RPLP2 HCC patients. (A–D) Kaplan–Meier curves indicating the DSS prognostic value of RPLP2 expression in different HCC subgroups including, Stage III and IV (A) (n of low = 44, n of high = 43), age ≤ 60 (B) (n of low = 87, n of high = 87), hepatocellular carcinoma (C) (n of low = 178, n of high = 177) and R0 (D) (n of low = 160, n of high = 160). (E–K) Kaplan–Meier curves indicating the PFI prognostic value of RPLP2 expression in different HCC subgroups including, N0 (E) (n of low = 127, n of high = 127), MO (F) (n of low = 134, n of high = 134), with tumor (G) (n of low = 76, n of high = 76), age ≤ 60 (H) (n of low = 88, n of high = 89), hepatocellular carcinoma (I) (n of low = 182, n of high = 181), R0 (J) (n of low = 163, n of high = 163) and G1 and G2 (K) (n of low = 116, n of high = 117). (The data was obtained from TCGA-LIHC). **Figure S4.** Forest map based on multivariate cox analysis for DSS and PFI. (A, B) Forest plots showing the potential prognostic indicators for DSS (A) and PFI (B). (The data was obtained from TCGA-LIHC). **Figure S5.** Calibration curves for predicting OS of HCC patients. (A–C) Calibration curve for predicting the 1- (A), 3- (B) and 5-year (C) overall survival rates of HCC patients. (The data was obtained from TCGA-LIHC). **Figure S6.** RPLP2-related DEGs and PPI network of these genes. (A) Volcano plot of DEGs (log-fold change > 1.5 and P < 0.01). (B) Heatmap of correlation between RPLP2 expression and top 10 RPLP2-related DEGs. (C) Interaction network of RPLP2-related DEGs in HCC via STRING. (D) Top 10 hub genes in RPLP2-related DEGs. (The data was obtained from TCGA-LIHC). **Figure S7.** Bioinformatics analysis of RPLP2’s role in ferroptosis in cancers other than HCC. (A) Correlation analysis of RPLP2 and GPX4 in CHOL, DLBC, GBM, LGG, PAAD, TGCT and THYM. (B) Gene set enrichment plots of “Oxidative Phosphorylation” from GSEA of RPLP2-related DEGs in GBM, LGG, PAAD, TGCT and THYM. (C) Gene set enrichment plots of “Lipid Catabolic Process” from GSEA of RPLP2-related DEGs in GBM, LGG, TGCT and THYM. (D) Gene set enrichment plots of “Cellular Iron Ion Homeostasis” from GSEA of RPLP2-related DEGs in THYM. (The data were obtained from TCGA-GBM, TCGA-LGG, TCGA-PAAD, TCGA-TGCT and TCGA-THYM). **Table S1.** Clinical features of 12 HCC patients. **Table S2.** Cox regression analyses of variables including RPLP2 level for OS in HCC patients. **Table S3.** Cox regression analyses of variables including RPLP2 level for DSS in HCC patients. **Table S4.** Cox regression analyses of variables including RPLP2 level for PFI in HCC patients.

## Data Availability

Correspondence and requests for materials or data should be addressed to Bokang Yan.
