# Supplementary material for: Highly expressed RPLP2 inhibits ferroptosis to promote hepatocellular carcinoma progression and predicts poor prognosis
Source: Cancer Cell Int. 2023 Nov 18;23:278. doi: 10.1186/s12935-023-03140-0 (PMC10656893; doi:10.1186/s12935-023-03140-0)
Supplement: Supplementary file 1 — Additional file 1: Figure S1. The expression level of RPLP2 in HCC and its localization in cancer. (A) RPLP2 mRNA expression level in peritumoral liver tissues and HCC tissues in GSE84402 database. (B) IHC test of RPLP2 protein expression in HCC. (C, D) Immunofluorescence assay of RPLP2 and microtubules in PC3 (C) and U2OS (D) cell lines from HPA database. *P < 0.05, ****P < 0.0001. Figure S2. Correlations between RPLP2 expression and molecular/immune subtypes in HCC. (A) Correlations between RPLP2 expression and molecular subtypes in HCC obtained from TISIDB database. (B) Correlations between RPLP2 expression and immune subtypes in HCC obtained from TISIDB database. Figure S3. DSS and PFI survival curve in different subgroups between high- and low-RPLP2 HCC patients. (A–D) Kaplan–Meier curves indicating the DSS prognostic value of RPLP2 expression in different HCC subgroups including, Stage III and IV (A) (n of low = 44, n of high = 43), age ≤ 60 (B) (n of low = 87, n of high = 87), hepatocellular carcinoma (C) (n of low = 178, n of high = 177) and R0 (D) (n of low = 160, n of high = 160). (E–K) Kaplan–Meier curves indicating the PFI prognostic value of RPLP2 expression in different HCC subgroups including, N0 (E) (n of low = 127, n of high = 127), MO (F) (n of low = 134, n of high = 134), with tumor (G) (n of low = 76, n of high = 76), age ≤ 60 (H) (n of low = 88, n of high = 89), hepatocellular carcinoma (I) (n of low = 182, n of high = 181), R0 (J) (n of low = 163, n of high = 163) and G1 and G2 (K) (n of low = 116, n of high = 117). (The data was obtained from TCGA-LIHC). Figure S4. Forest map based on multivariate cox analysis for DSS and PFI. (A, B) Forest plots showing the potential prognostic indicators for DSS (A) and PFI (B). (The data was obtained from TCGA-LIHC). Figure S5. Calibration curves for predicting OS of HCC patients. (A–C) Calibration curve for predicting the 1- (A), 3- (B) and 5-year (C) overall survival rates of HCC patients. (The data was o [file 12935_2023_3140_MOESM1_ESM.docx]

Additional file 1 for

**Highly expressed RPLP2 inhibits ferroptosis to promote hepatocellular carcinoma progression and predicts poor prognosis**

Jiaxing Guo^1^, Meiyuan Huang^2^, Shuang Deng^2^, Haiyan Wang^3^, Zuli Wang^4^, Bokang Yan^2^*

*Correspondence: Bokang Yan (bokangyan2022@163.com)

**This file includes:**

Figure S1 to S7

Table S1 to S4


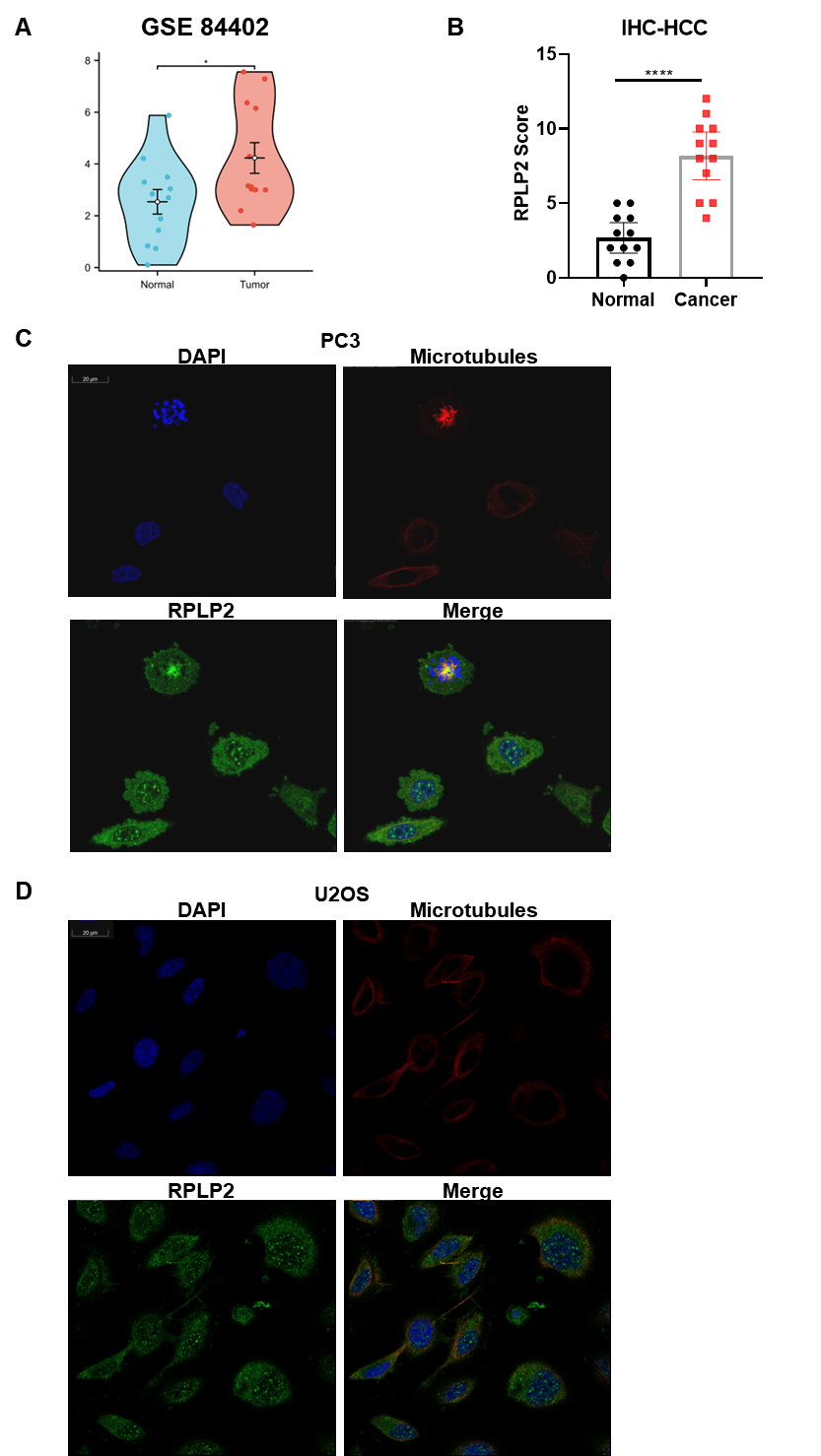


**Figure S1. The expression level of RPLP2 in HCC and its localization in cancer.** (A) RPLP2 mRNA expression level in peritumoral liver tissues and HCC tissues in GSE84402 database. (B) IHC test of RPLP2 protein expression in HCC. (C, D) Immunofluorescence assay of RPLP2 and microtubules in PC3 (C) and U2OS (D) cell lines from HPA database. * P<0.05, **** P<0.0001.

**
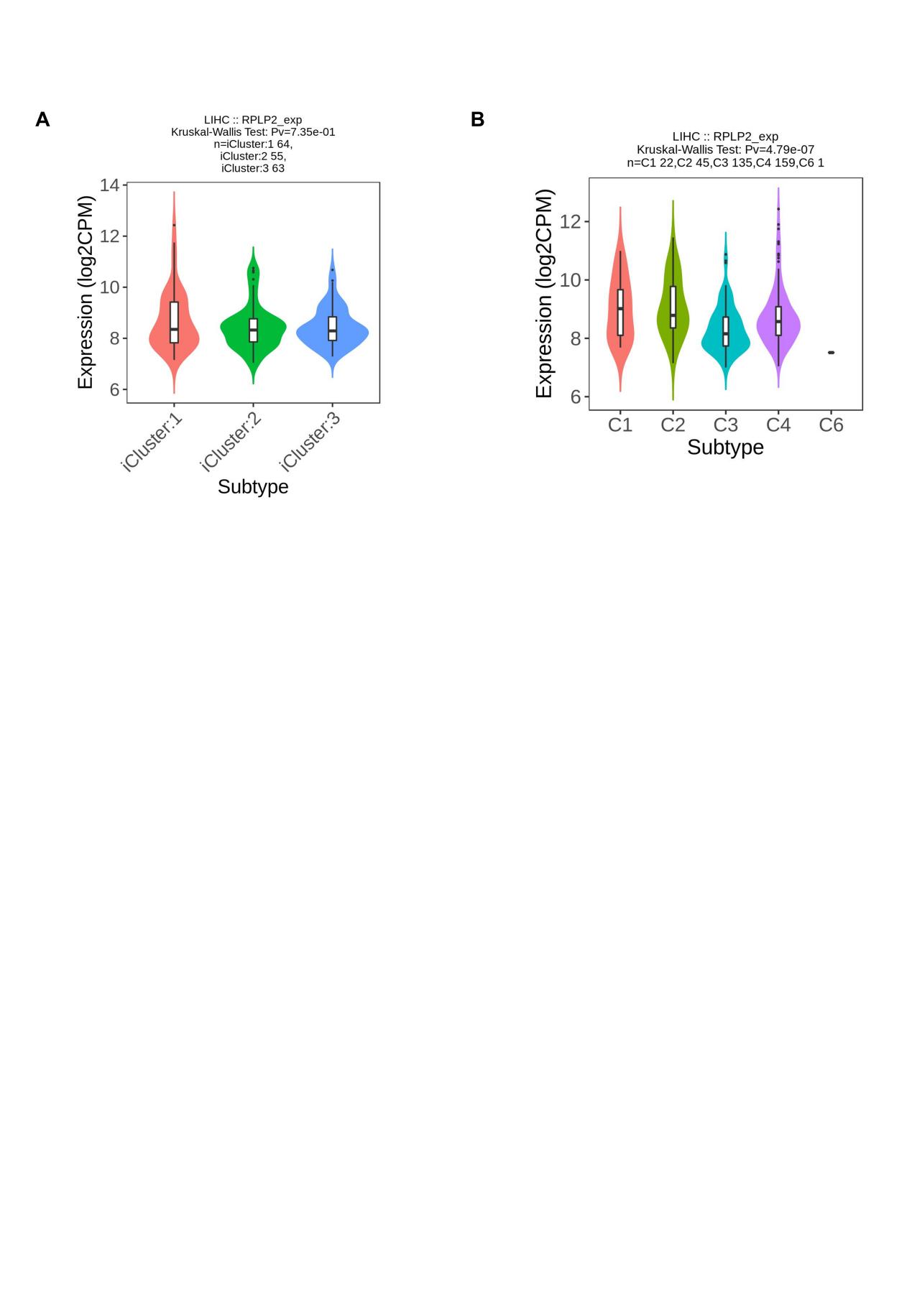
Figure S2. Correlations between RPLP2 expression and molecular / immune subtypes in HCC.** (A) Correlations between RPLP2 expression and molecular subtypes in HCC obtained from TISIDB database. (B) Correlations between RPLP2 expression and immune subtypes in HCC obtained from TISIDB database.


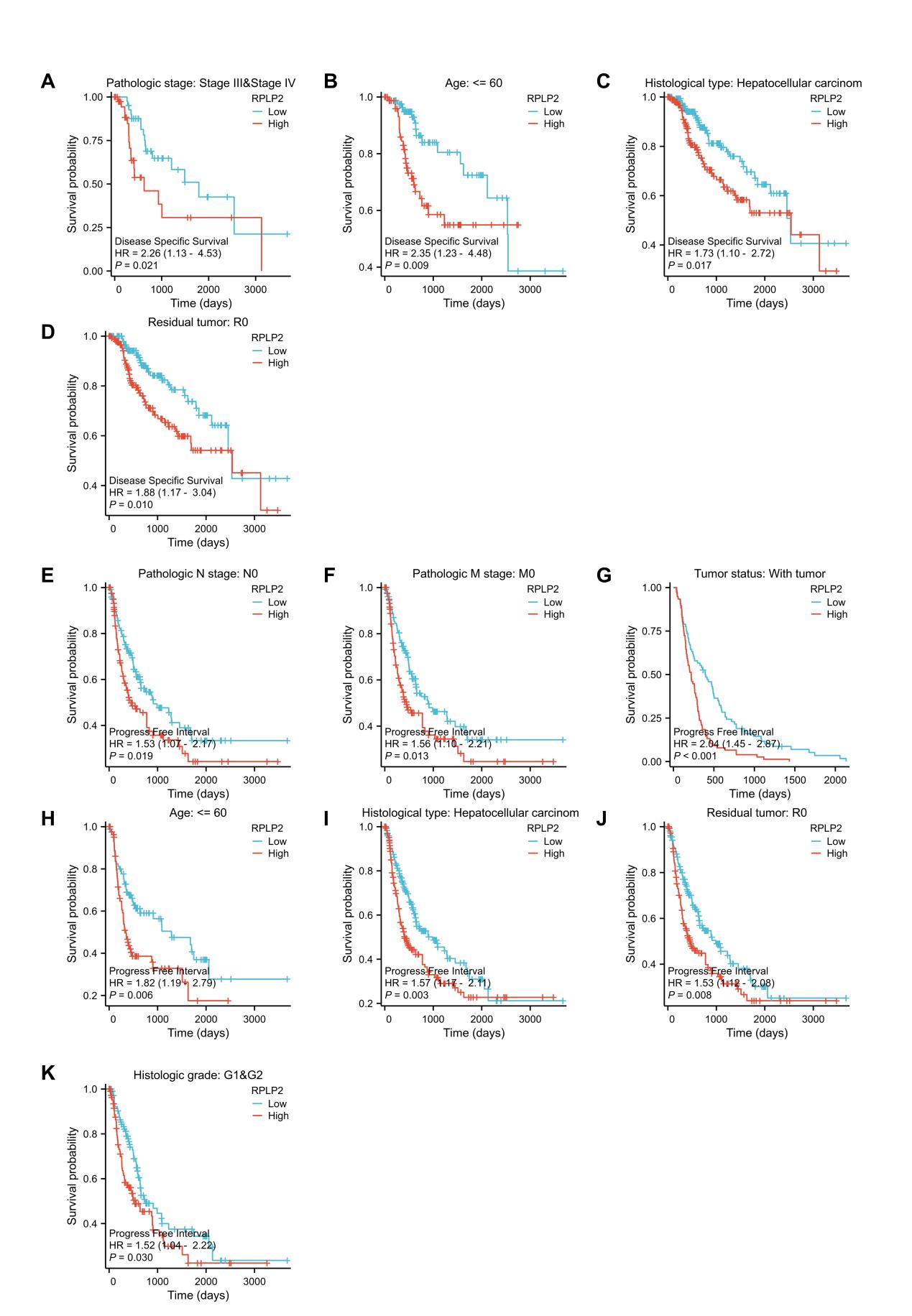


**Figure S3. DSS and PFI survival curve in different subgroups between high- and low-RPLP2 HCC patients.** (A-D) Kaplan-Meier curves indicating the DSS prognostic value of RPLP2 expression in different HCC subgroups including, Stage III and IV (A) (n of low=44, n of high=43), age ≤ 60 (B) (n of low=87, n of high=87), hepatocellular carcinoma (C) (n of low=178, n of high=177) and R0 (D) (n of low=160, n of high=160). (E-K) Kaplan-Meier curves indicating the PFI prognostic value of RPLP2 expression in different HCC subgroups including, N0 (E) (n of low=127, n of high=127), MO (F) (n of low=134, n of high=134), with tumor (G) (n of low=76, n of high=76), age ≤ 60 (H) (n of low=88, n of high=89), hepatocellular carcinoma (I) (n of low=182, n of high=181), R0 (J) (n of low=163, n of high=163) and G1 and G2 (K) (n of low=116, n of high=117). (The data was obtained from TCGA-LIHC.)


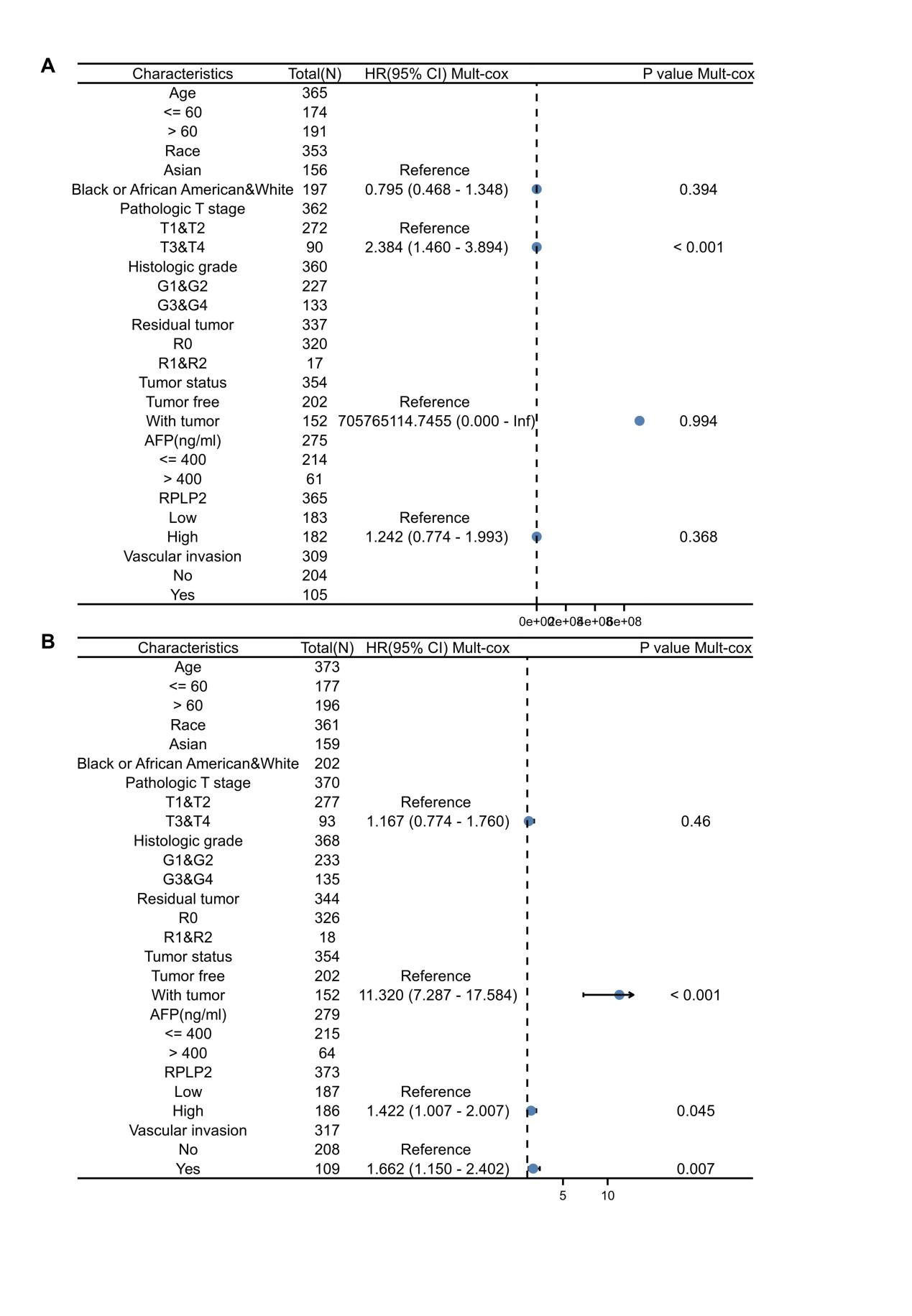


**Figure S4. Forest map based on multivariate cox analysis for DSS and PFI.** (A, B) Forest plots showing the potential prognostic indicators for DSS (A) and PFI (B). (The data was obtained from TCGA-LIHC.)

**
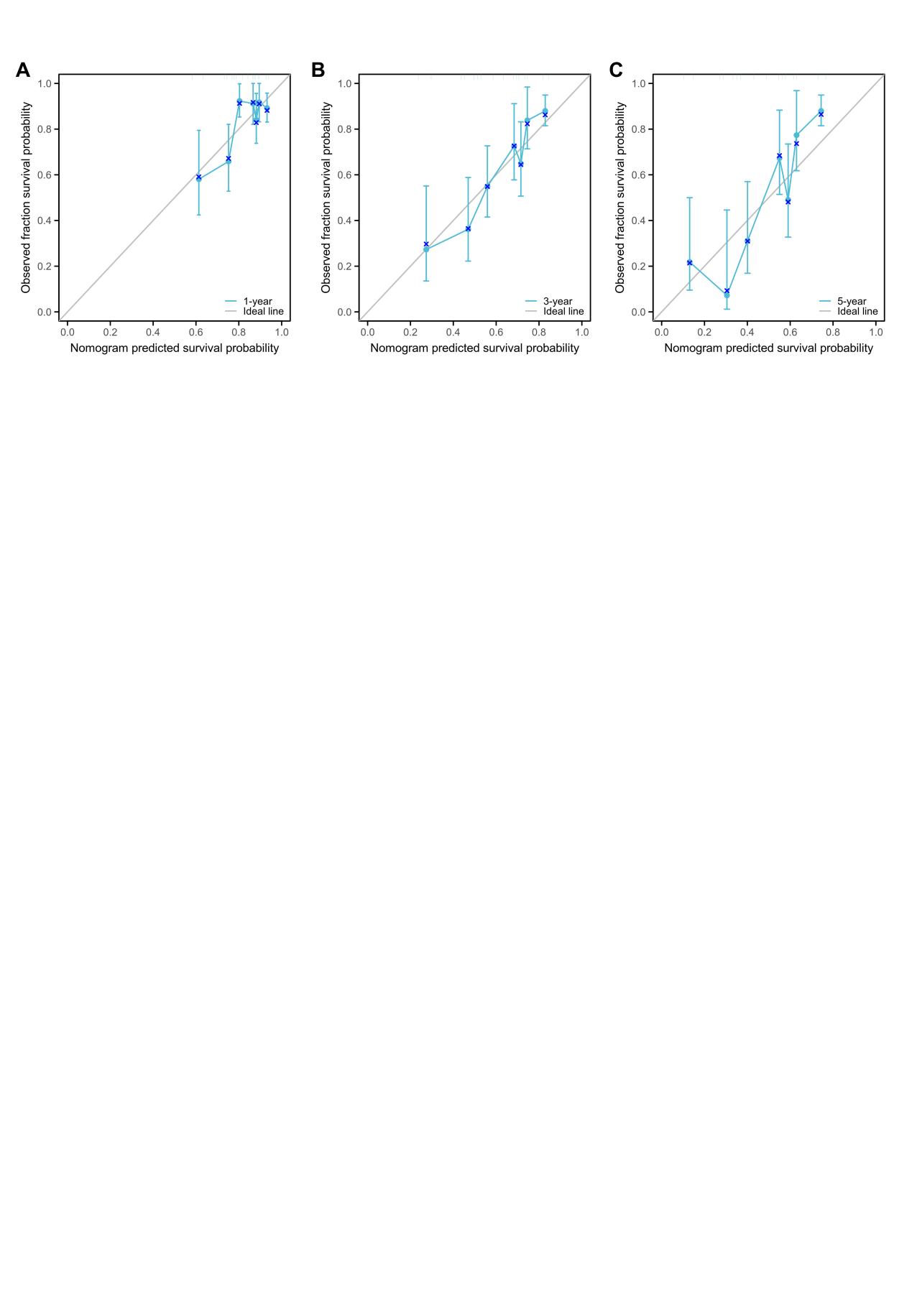
Figure S5. Calibration curves for predicting OS of HCC patients.** (A-C) Calibration curve for predicting the 1- (A), 3- (B) and 5-year (C) overall survival rates of HCC patients. (The data was obtained from TCGA-LIHC.)


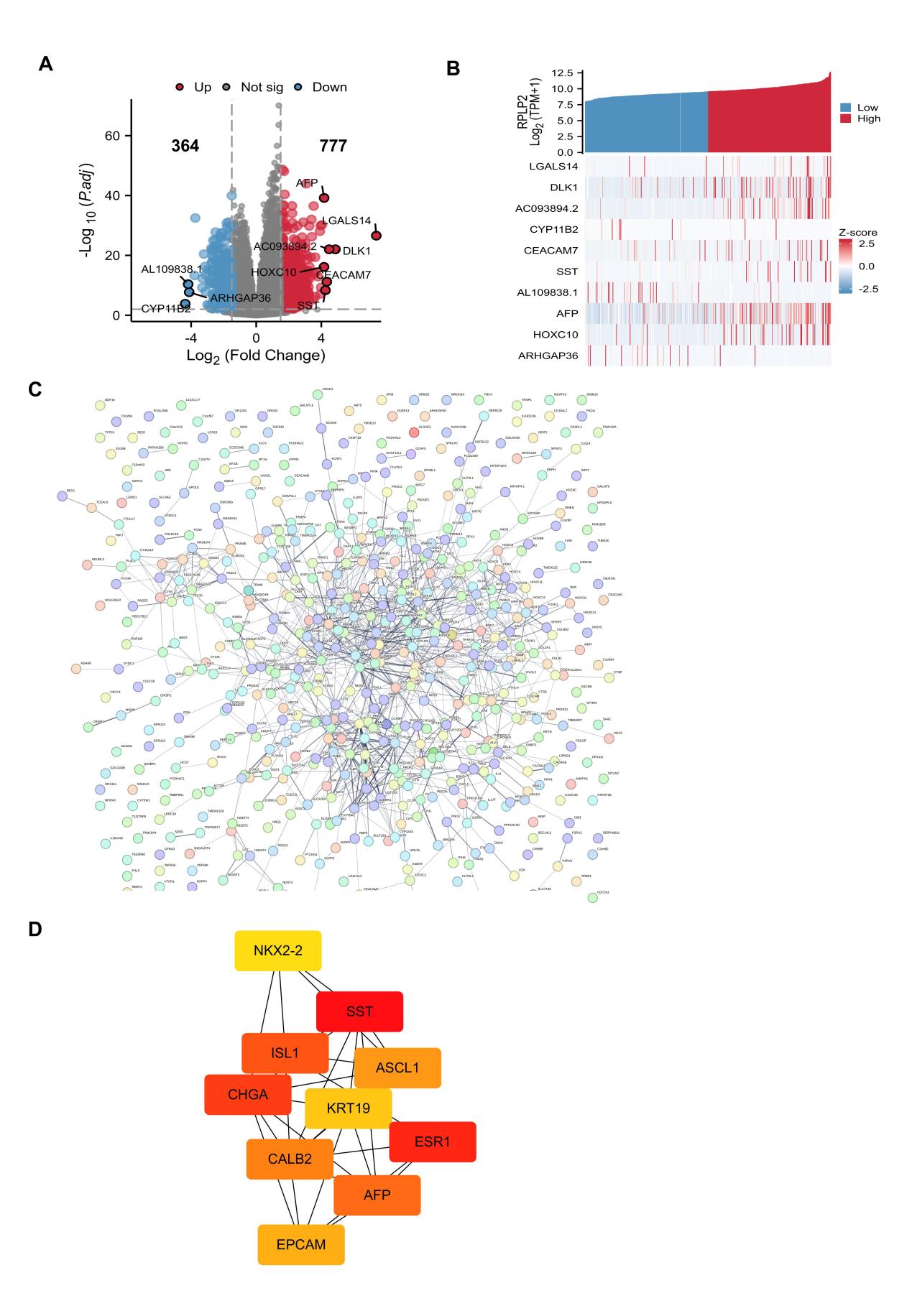


**Figure S6. RPLP2-related DEGs and PPI network of these genes.** (A) Volcano plot of DEGs (log-fold change >1.5 and p < 0.01). (B) Heatmap of correlation between RPLP2 expression and top 10 RPLP2-related DEGs. (C) Interaction network of RPLP2-related DEGs in HCC via STRING. (D) Top 10 hub genes in RPLP2-related DEGs. (The data was obtained from TCGA-LIHC.)


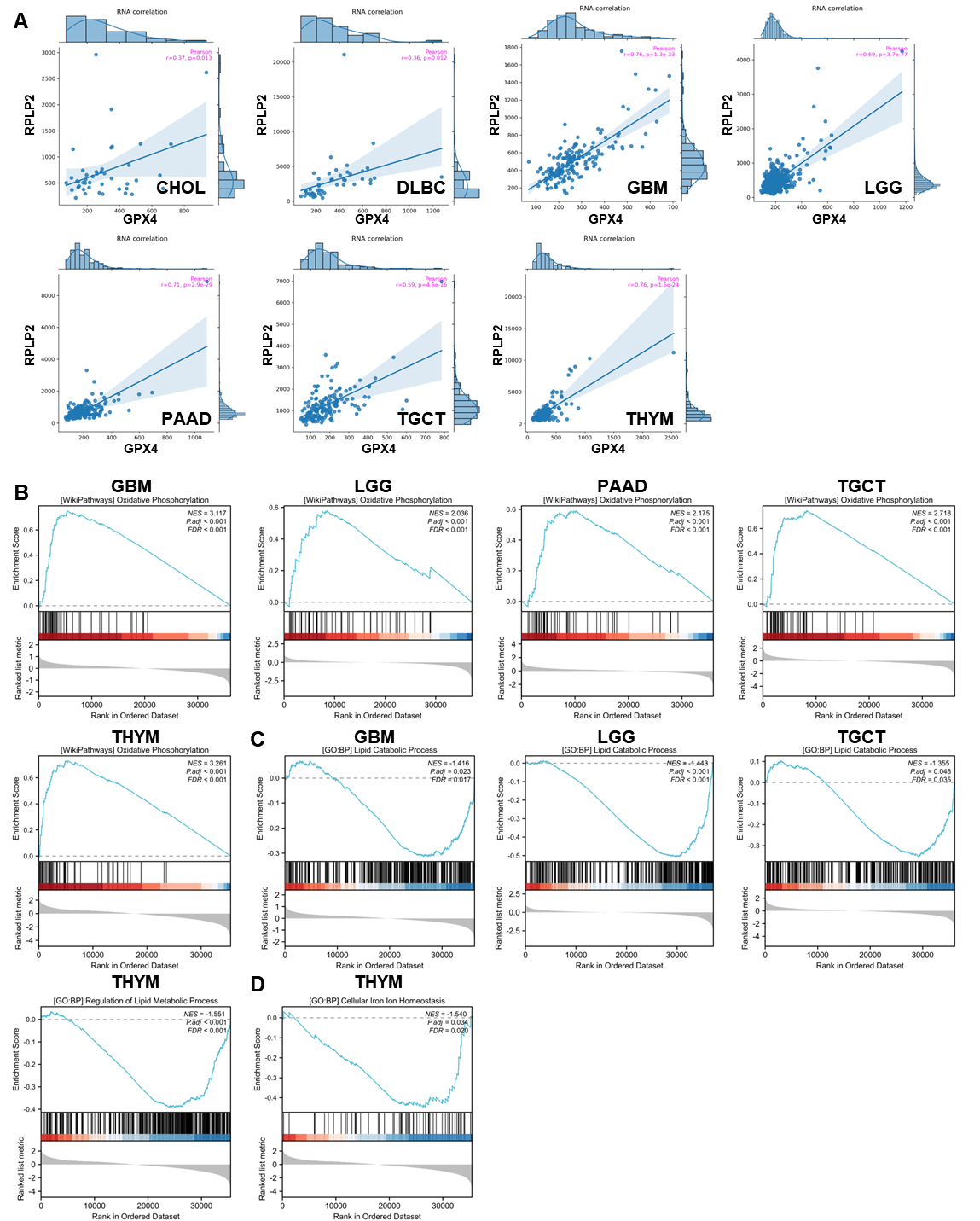


**Figure S7. Bioinformatics analysis of RPLP2’s role in ferroptosis in cancers other than HCC.** (A) Correlation analysis of RPLP2 and GPX4 in CHOL, DLBC, GBM, LGG, PAAD, TGCT and THYM. (B) Gene set enrichment plots of “Oxidative Phosphorylation” from GSEA of RPLP2-related DEGs in GBM, LGG, PAAD, TGCT and THYM. (C) Gene set enrichment plots of “Lipid Catabolic Process” from GSEA of RPLP2-related DEGs in GBM, LGG, TGCT and THYM. (D) Gene set enrichment plots of “Cellular Iron Ion Homeostasis” from GSEA of RPLP2-related DEGs in THYM. (The data were obtained from TCGA-GBM, TCGA-LGG, TCGA-PAAD, TCGA-TGCT and TCGA-THYM)

**Table S1. Clinical features of 12 HCC patients.**

| Gender | Age | T stage | N stage | M stage | Pathologic stage | Level of diferentiation | AFP  (ng/ml) | R0 resection | RPLP2 expression |
| --- | --- | --- | --- | --- | --- | --- | --- | --- | --- |
| male | 54 | T1 | N0 | M0 | I | Well | 78.2 | Yes | 8 |
| female | 67 | T3 | N0 | M0 | II | Well | 255.9 | Yes | 9 |
| male | 65 | T1 | N0 | M0 | I | Middle | 247.8 | Yes | 5 |
| female | 52 | T2 | N0 | M0 | I | Middle | 387.5 | Yes | 7 |
| male | 78 | T2 | N1 | M0 | III | Poor | 568.2 | Yes | 9 |
| male | 57 | T1 | N0 | M0 | I | Well | 145.3 | Yes | 4 |
| female | 82 | T4 | N0 | M1 | IV | Poor | 512.3 | No | 11 |
| female | 59 | T3 | N0 | M0 | II | Middle | 644.1 | No | 10 |
| male | 61 | T3 | N1 | M0 | III | Poor | 844.1 | Yes | 10 |
| female | 72 | T3 | N1 | M0 | III | Well | 503.5 | Yes | 5 |
| male | 81 | T3 | N0 | M0 | II | Poor | 412.4 | Yes | 12 |
| female | 64 | T3 | N0 | M0 | II | Middle | 473.9 | Yes | 8 |

**Table S2. Cox regression analyses of variables including RPLP2 level for OS in HCC patients.**

| Characteristics | Total(N) | Univariate analysis | |  | Multivariate analysis | |
| --- | --- | --- | --- | --- | --- | --- |
|  |  | Hazard ratio (95% CI) | P value |  | Hazard ratio (95% CI) | P value |
| Age | 373 |  | 0.293 |  |  |  |
| <= 60 | 177 | Reference |  |  |  |  |
| > 60 | 196 | 1.205 (0.850 - 1.708) | 0.295 |  |  |  |
| Race | 361 |  | 0.117 |  |  |  |
| Asian | 159 | Reference |  |  |  |  |
| Black or African American&White | 202 | 1.341 (0.926 - 1.942) | 0.121 |  |  |  |
| Pathologic T stage | 370 |  | **< 0.001** |  |  |  |
| T1&T2 | 277 | Reference |  |  | Reference |  |
| **T3&T4** | 93 | 2.598 (1.826 - 3.697) | **< 0.001** |  | 2.267 (1.557 - 3.302) | **< 0.001** |
| Histologic grade | 368 |  | 0.637 |  |  |  |
| G1&G2 | 233 | Reference |  |  |  |  |
| G3&G4 | 135 | 1.091 (0.761 - 1.564) | 0.636 |  |  |  |
| Residual tumor | 344 |  | 0.203 |  |  |  |
| R0 | 326 | Reference |  |  |  |  |
| R1&R2 | 18 | 1.604 (0.812 - 3.169) | 0.174 |  |  |  |
| Tumor status | 354 |  | **< 0.001** |  |  |  |
| Tumor free | 202 | Reference |  |  | Reference |  |
| **With tumor** | 152 | 2.317 (1.590 - 3.376) | **< 0.001** |  | 1.851 (1.255 - 2.731) | **0.002** |
| AFP(ng/ml) | 279 |  | 0.773 |  |  |  |
| <= 400 | 215 | Reference |  |  |  |  |
| > 400 | 64 | 1.075 (0.658 - 1.759) | 0.772 |  |  |  |
| RPLP2 | 373 |  | **0.001** |  |  |  |
| Low | 187 | Reference |  |  | Reference |  |
| **High** | 186 | 1.791 (1.258 - 2.548) | **0.001** |  | 1.718 (1.185 - 2.491) | **0.004** |
| Vascular invasion | 317 |  | 0.169 |  |  |  |
| No | 208 | Reference |  |  |  |  |
| Yes | 109 | 1.344 (0.887 - 2.035) | 0.163 |  |  |  |

**Table S3. Cox regression analyses of variables including RPLP2 level for DSS in HCC patients.**

| Characteristics | Total(N) | Univariate analysis | |  | Multivariate analysis | |
| --- | --- | --- | --- | --- | --- | --- |
|  |  | Hazard ratio (95% CI) | P value |  | Hazard ratio (95% CI) | P value |
| Age | 365 |  | 0.459 |  |  |  |
| <= 60 | 174 | Reference |  |  |  |  |
| > 60 | 191 | 0.846 (0.543 - 1.317) | 0.458 |  |  |  |
| Race | 353 |  | 0.092 |  |  |  |
| Asian | 156 | Reference |  |  | Reference |  |
| Black or African American&White | 197 | 1.500 (0.927 - 2.427) | 0.098 |  | 0.795 (0.468 - 1.348) | 0.394 |
| Pathologic T stage | 362 |  | **< 0.001** |  |  |  |
| T1&T2 | 272 | Reference |  |  | Reference |  |
| **T3&T4** | 90 | 3.639 (2.328 - 5.688) | **< 0.001** |  | 2.384 (1.460 - 3.894) | **< 0.001** |
| Histologic grade | 360 |  | 0.727 |  |  |  |
| G1&G2 | 227 | Reference |  |  |  |  |
| G3&G4 | 133 | 1.086 (0.683 - 1.728) | 0.726 |  |  |  |
| Residual tumor | 337 |  | 0.258 |  |  |  |
| R0 | 320 | Reference |  |  |  |  |
| R1&R2 | 17 | 1.678 (0.728 - 3.870) | 0.224 |  |  |  |
| Tumor status | 354 |  | **< 0.001** |  |  |  |
| Tumor free | 202 | Reference |  |  | Reference |  |
| With tumor | 152 | 775790759.3892 (0.000 - Inf) | 0.994 |  | 705765114.7455 (0.000 - Inf) | 0.994 |
| AFP(ng/ml) | 275 |  | 0.665 |  |  |  |
| Characteristics | Total(N) | Univariate analysis | |  | Multivariate analysis | |
|  |  | Hazard ratio (95% CI) | P value |  | Hazard ratio (95% CI) | P value |
| <= 400 | 214 | Reference |  |  |  |  |
| > 400 | 61 | 0.867 (0.450 - 1.668) | 0.668 |  |  |  |
| RPLP2 | 365 |  | **0.029** |  |  |  |
| Low | 183 | Reference |  |  | Reference |  |
| High | 182 | 1.641 (1.049 - 2.569) | **0.030** |  | 1.242 (0.774 - 1.993) | 0.368 |
| Vascular invasion | 309 |  | 0.425 |  |  |  |
| No | 204 | Reference |  |  |  |  |
| Yes | 105 | 1.277 (0.707 - 2.306) | 0.418 |  |  |  |

**Table S4. Cox regression analyses of variables including RPLP2 level for PFI in HCC patients.**

| Characteristics | Total(N) | Univariate analysis | |  | Multivariate analysis | |
| --- | --- | --- | --- | --- | --- | --- |
|  |  | Hazard ratio (95% CI) | P value |  | Hazard ratio (95% CI) | P value |
| Age | 373 |  | 0.783 |  |  |  |
| <= 60 | 177 | Reference |  |  |  |  |
| > 60 | 196 | 0.960 (0.718 - 1.284) | 0.783 |  |  |  |
| Race | 361 |  | 0.194 |  |  |  |
| Asian | 159 | Reference |  |  |  |  |
| Black or African American&White | 202 | 1.218 (0.903 - 1.643) | 0.196 |  |  |  |
| Pathologic T stage | 370 |  | **< 0.001** |  |  |  |
| T1&T2 | 277 | Reference |  |  | Reference |  |
| T3&T4 | 93 | 2.177 (1.590 - 2.980) | **< 0.001** |  | 1.167 (0.774 - 1.760) | 0.460 |
| Histologic grade | 368 |  | 0.357 |  |  |  |
| G1&G2 | 233 | Reference |  |  |  |  |
| G3&G4 | 135 | 1.152 (0.853 - 1.557) | 0.355 |  |  |  |
| Residual tumor | 344 |  | 0.193 |  |  |  |
| R0 | 326 | Reference |  |  |  |  |
| R1&R2 | 18 | 1.513 (0.840 - 2.726) | 0.168 |  |  |  |
| Tumor status | 354 |  | **< 0.001** |  |  |  |
| Tumor free | 202 | Reference |  |  | Reference |  |
| **With tumor** | 152 | 11.342 (7.567 - 17.000) | **< 0.001** |  | 11.320 (7.287 - 17.584) | **< 0.001** |
| AFP(ng/ml) | 279 |  | 0.832 |  |  |  |
| <= 400 | 215 | Reference |  |  |  |  |
| > 400 | 64 | 1.045 (0.698 - 1.563) | 0.832 |  |  |  |
| RPLP2 | 373 |  | **0.003** |  |  |  |
| Low | 187 | Reference |  |  | Reference |  |
| **High** | 186 | 1.554 (1.161 - 2.081) | **0.003** |  | 1.422 (1.007 - 2.007) | **0.045** |
